# Sudden death of quantum advantage in correlation generations

**DOI:** 10.1126/sciadv.adr5002

**Published:** 2024-11-22

**Authors:** Weixiao Sun, Fuchuan Wei, Yuguo Shao, Zhaohui Wei

**Affiliations:** ^1^ Institute for Interdisciplinary Information Sciences, Tsinghua University, Beijing 100084, China.; ^2^ Yau Mathematical Sciences Center, Tsinghua University, Beijing 100084, China.; ^3^ Department of Mathematics, Tsinghua University, Beijing 100084, China.; ^4^ Yanqi Lake Beijing Institute of Mathematical Sciences and Applications, Beijing 101407, China.

## Abstract

Quantum noise is one of the most profound obstacles to implementing large-scale quantum algorithms and schemes. In particular, the dynamical process by which quantum noise, varying in strength from 0 to critical levels, affects and destroys quantum advantage has not been well understood. Meanwhile, correlation generation serves as a precious theoretical model for information processing tasks, where quantum advantage can be precisely quantified. In this study, we show that this model provides valuable insights into the understanding of this dynamical process. We prove that, as the strength of quantum noise continuously increases from 0, the quantum advantage diminishes gradually and eventually vanishes. Unexpectedly, in some cases, we observe the phenomenon of a sudden death of quantum advantage: When the noise strength exceeds a certain threshold, the quantum advantage abruptly disappears from a substantial level. This phenomenon, once again, reveals the tremendous impact of noise on quantum information processing tasks.

## Teaser

The dynamic process by which quantum noise, ranging from 0 to critical levels, affects quantum advantage is characterized.

## INTRODUCTION

Quantum noise is one of the major obstacles in building large-scale quantum computers. Although remarkable progress in their physical implementation has been made (*1*–*5*), we still lack sufficient quantum computational resources to perform quantum error corrections, which are considered the ultimate approach to combat quantum noise. As a result, we are now limited to working with noisy intermediate-scale quantum (NISQ) computers (*6*–*8*), which usually based on short-depth quantum circuits that do not incorporate error correction. In recent years, researchers have intensively used NISQ computers to sample outputs from random quantum circuits and solve optimization problems such as calculating the ground-state energy of physically relevant Hamiltonians, in an effort to demonstrate that these computers can efficiently solve problems that classical computers cannot. To reliably exhibit quantum advantage, it is crucial not only to improve the quality of quantum hardware and mitigate experimental imperfections for higher precision but also to understand how noise affects the computational advantage of quantum computing. This paper focuses on the latter aspect.

Addressing the impact of noise on quantum advantage presents two major challenges. First, rigorous characterization of quantum advantage is usually difficult to achieve. For example, while Shor’s algorithm marked a milestone in quantum computing, it remains unproven whether it can achieve an exponential speedup over the best classical counterpart (*9*), as the classical computational complexity of integer factorization is still unresolved. In some specific computational models, such as query complexity (*10*, *11*), communication complexity (*12*, *13*), and certain machine learning models (*14*, *15*), researchers have mathematically demonstrated quantum advantage, often by providing rough upper bounds on the power of quantum computing while simultaneously lower bounding the classical counterpart’s performance on the same task. However, after factoring in the impact of quantum noise, it becomes exceedingly difficult to quantify quantum advantage for most problems. Therefore, to investigate the impacts of noise of varying strengths on quantum advantage, a proper measure for quantifying it is essential.

Second, the influence of noise on quantum circuits is highly complex and not yet well understood. In recent years, spurred by the impact of quantum supremacy experiments based on random quantum circuits (*1*–*3*), substantial efforts have been devoted to analyzing the properties of noisy random quantum circuits (*16*–*30*). Because quantum advantage is achieved only when a quantum computer can solve a computational problem more efficiently than classical computers, a key focus of this research is determining when sampling from the outputs of noisy random quantum circuits can or cannot be efficiently simulated by classical algorithms. For example, it has been proven that, with a constant noise rate per quantum gate, sampling from the output distribution of a noisy random circuit can be approximately simulated by an efficient classical algorithm (*21*), assuming the anti-concentration property (*31*). This unexpected result highlights the importance of further reducing error rates in NISQ computers. In (*26*), strong evidence was provided showing that, under certain conditions, local errors in random quantum circuits are scrambled and can be approximated by global white noise, which justifies an assumption critical to Google’s quantum supremacy experiment (*1*, *18*). Meanwhile, when demonstrating quantum supremacy with random quantum circuits, a substantial challenge lies in verifying the existence of quantum advantage at a reasonable experimental cost. To address this, several verification tools, such as the heavy-outcome generation fidelity (*16*) and the linear cross-entropy benchmark (*1*), have been proposed and extensively studied (*27*–*29*).

In addition to sampling the outputs of random quantum circuits, recent studies have also investigated the quantum supremacy of NISQ computers in solving optimization problems (*32*–*34*). These works suggest that quantum advantage is achievable only when the error rate is exceptionally low. Please see (*32*–*41*) for more discussions on how noise affects quantum computing.

Despite these promising advances, in most cases, the quantum advantage of noisy NISQ computers can only be identified in small or limited areas of the parameter spaces of quantum circuits, such as on one side of specific transition points (*22*, *42*, *43*). As a result, the step-by-step dynamical process through which quantum noise, continuously increasing in strength from 0 to a critical level, gradually diminishes and eventually destroys quantum advantage remains poorly understood. Gaining a deeper understanding of this process is important for the development of NISQ computers capable of achieving practical quantum supremacy.

Here, we focus on a simpler sampling model than that of sampling from the outputs of noisy random quantum circuits. In our setting, two separate parties, Alice and Bob, cooperate to sample two separated outcomes according to a target joint probability distribution (*44*, *45*). This task can be accomplished using either a quantum or a classical scheme. In the quantum approach, Alice and Bob locally measure a shared quantum seed state without any communication and then output the corresponding outcomes. In the classical approach, they replace the shared quantum state with public randomness, using only local classical operations for further actions. A crucial feature of this elegant model is that a quantum scheme can achieve a remarkable advantage over any classical one. Furthermore, in the noiseless case, the minimum computational costs for both scenarios, measured by the sizes of the shared quantum states and the public randomness, can be exactly characterized (*44*, *45*). This precise quantification of quantum advantage makes the model particularly valuable. On the basis of this fact, we show that this model allows us to rigorously analyze the dynamical process by which noise of increasing strength affects quantum advantage. Specifically, for any given target correlation, we first define a measure of quantum advantage within this model. We then prove that, as the strength of noise continuously increases from zero, the quantum advantage gradually diminishes and eventually disappears. Unexpectedly, we also report a phenomenon that we call the sudden death of quantum advantage, where, in some cases, once the noise reaches a certain threshold, the quantum advantage abruptly vanishes from a substantial level. This intriguing phenomenon once again highlights the detrimental effects of noise in quantum information processing tasks.

## RESULTS

### Setting

Suppose that two separate parties, Alice and Bob, aim to sample two outcomes, *x* ∈ [*m*] ≡ {1,2,…, *m*} and *y* ∈ [*n*], respectively, according to a discrete joint probability distribution *P* = [*P*(*x*, *y*)]_*x*∈[*m*],*y*∈[*n*]_. In other words, the probability that Alice outputs *x* and Bob outputs *y* is *P*(*x*, *y*). If this is the case, then we say that Alice and Bob generate *P*, and *P* is referred to as a classical correlation. We can represent *P* as an *m* × *n* nonnegative matrix, a matrix with all entries nonnegative. Without loss of generality, we assume for simplicity that *m* = *n* in this work. When generating *P*, we assume that no communication is allowed between Alice and Bob. If the target *P* is not a product distribution, that is, rank(*P*) > 1, then they will need to share some initial resource to generate *P*. If Alice and Bob choose to generate *P* using a classical protocol, then the initial shared resource can be another classical correlation *P*′, known as a seed correlation. In this case, Alice and Bob perform local classical operations on *P*′ to generate *P*. Similarly, they can generate *P* using a quantum protocol, in which the initial shared resource is a shared quantum state ρ, called a seed state. Alice and Bob then perform appropriate local positive-operator–valued measurements (POVMs) on ρ, and output the corresponding outcomes to obtain *P*. We define the size of *P*′ or ρ as half of the total number of bits or qubits required to represent *P*′ or ρ and denote this as size(*P*′) or size(ρ), respectively. For more details on this model of correlation generation, please refer to (*44*, *45*).

For a given target *P*, we are concerned with the minimum value of size(*P*′) [or size(ρ)] for *P*′ (or ρ) that can generate *P*. This value is known as the classical (quantum) correlation complexity of *P*, denoted by R(*P*) [or Q(*P*)], respectively. These complexities have been fully characterized (*44*, *45*). Specifically, it holds that

$$R\left(P\right)=\left\lceil {\text{log}}_{2}{\text{rank}}_{+}\left(P\right)\right\rceil$$ (1)

and

$$Q\left(P\right)=\left\lceil {\text{log}}_{2}{\text{rank}}_{\text{psd}}\left(P\right)\right\rceil$$ (2)

For a nonnegative matrix $P\in {\mathbb{R}}_{\geq 0}^{n\times n}$, rank_+_(*P*) is the nonnegative rank, defined as the minimum integer *r* such that *P* can be expressed as the sum of *r* nonnegative rank-1 matrices. rank_psd_(*P*) is the positive semidefinite (PSD) rank, defined as the minimum integer *r* such that there exist *r* × *r* PSD matrices *C_x_*, *D_y_* ∈ ℂ^*r*×*r*^ satisfying *P*(*x*, *y*) = Tr(*C_x_D_y_*) for all *x* and *y*. This factorization is called a PSD decomposition (*46*, *47*). Although both ranks are NP-hard to compute (*48*, *49*), this problem provides a valuable model in which quantum advantage can be precisely quantified. It has been shown that there exists a classical correlation $P\in {\mathbb{R}}_{\geq 0}^{2^{n}\times 2^{n}}$ such that rank_+_(*P*) = 2^Ω(*n*)^, while rank_psd_(*P*) = *O*(*n*) (*46*). This implies that quantum schemes can have a remarkable advantage over classical ones in generating correlations.

In the quantum protocol, however, the states shared by Alice and Bob may suffer from noise because of various experimental imperfections, which can substantially affect the quantum advantage described above. Analyzing this impact is the main motivation of this paper. For simplicity, we assume that all quantum noise is concentrated on the preparation and quantum memory of the initial seed states, while all other quantum operations are assumed to be noiseless. For example, a noisy POVM can be modeled as a noise channel combined with the ideal POVM. The POVM itself can then be regarded as noiseless if the noise channel is merged with the operations preceding the POVM. Meanwhile, we assume that classical protocols remain noiseless throughout. Specifically, in a quantum protocol, we assume that the seed state ρ is affected by bipartite quantum noise of the form E_λ_ ⊗ E_λ_, meaning each subsystem of ρ passes through a global depolarizing noise channel E_λ_ with strength λ. This channel leaves the subsystems unchanged with probability 1 − λ and replaces them with the maximally mixed state with probability λ. It is important to note that this type of noise can naturally occur in real-life quantum systems. For example, Dalzell *et al*. (*26*) shows that, under certain conditions, the overall effect of local quantum errors in random quantum circuits can be approximated by global white noise. When the noise strength λ = 0, a quantum scheme for generating correlations may exhibit a remarkable advantage over classical counterparts. When λ = 1, the initial seed state fully degrades into white noise, making any quantum advantage impossible, as Alice and Bob can only produce a product distribution. In the remainder of this paper, we will characterize the process by which the quantum advantage diminishes as the noise strength λ increases from 0 to 1.

If a classical correlation *P* can be generated by ρ under the noise channel E_λ_ ⊗ E_λ_, we denote this as $\rho \overset{\lambda }{\to }P$. Under such a noise channel, we define the quantum advantage in generating the classical correlation *P*, denoted $S_{\lambda }\left(P\right)$, as the ratio between the lowest classical cost of generating *P* and the smallest size of ρ such that $\rho \overset{\lambda }{\to }P$. Formally

$$S_{\lambda }\left(P\right)=\frac{R\left(P\right)}{{\text{inf}}_{\rho }\text{size}\left(\rho \right)}$$ (3)

where the infimum is taken over all ρ such that $\rho \overset{\lambda }{\to }P$. This definition aligns with our intuition about quantum advantage: If $S_{\lambda }\left(P\right)$ is very large, e.g., 10, then it means that the classical cost of generating *P* is at least 9 times higher than that of the optimal noisy quantum protocol affected by the noise channel E_λ_ ⊗ E_λ_.

Before proceeding, we first analyze the quantum advantage of noiseless quantum protocols in generating classical correlations. It has been established that, for any arbitrary classical correlation *P*, the minimum quantum seed required to generate *P* can always be chosen as a pure quantum state (*50*). The following conclusion shows that noiseless quantum protocols offer a remarkable quantum advantage in generating classical correlations.

**Theorem 1** Suppose ρ = ∣ψ〉〈ψ∣ is a bipartite entangled pure quantum state on H*_A_* ⊗ H*_B_*. Then, there always exists a family of classical correlations ${\left\{P_{m}\right\}}_{{m\in \mathbb{Z}}^{+}}$ such that $\rho \overset{0}{\to }P_{m}$ for all m, and $\underset{m\to \infty }{\text{lim}}S_{0}\left(P_{m}\right)=\infty$.

The proof for Theorem 1 can be found in section S1. Here, we outline the main idea of the proof. For *m* distinct real numbers α_1_,…, α*_m_*, we define an *m* × *m* Euclidean distance matrix EDM*_m_* by ${\text{EDM}}_{m}\left(x,y\right)=\frac{{\left(\alpha_{x}-\alpha_{y}\right)}^{2}}{\sum_{\mathrm{ij}}{\left(\alpha_{i}-\alpha_{j}\right)}^{2}}$. It is known that rank_psd_(EDM*_m_*) = 2 and ${\text{rank}}_{+}({\text{EDM}}_{m})\geq 2\sqrt{m}-2$ (*51*). We then define a modified Euclidean distance matrix ${\text{EDM}}_{m}^{\prime }\equiv L\cdot {\text{EDM}}_{m}\cdot R$, where *L* and *R* are two *m* × *m* diagonal matrices with positive diagonal entries. We can prove that, for any bipartite entangled pure state ∣ψ〉 and any integer *m*, ∣ψ〉 can always generate a classical correlation *P_m_* such that some submatrix of *P_m_* is an *m* × *m* modified Euclidean distance matrix ${\text{EDM}}_{m}^{\prime }$. It follows that $S_{0}\left(P_{m}\right)\geq \left\lceil {\text{log}}_{2}\left(2\sqrt{m}-2\right)\right\rceil /\text{size}\left(\rho \right)=\Omega \left(\text{log}\left(m\right)\right)$. Because *m* can be chosen arbitrarily, the proof is complete.

We now turn to the case of noisy quantum protocols that generate classical correlations and explore how noise affects quantum advantage.

### Case of strong noise: Destruction of reachability

Let us first consider the scenario of strong quantum noise. Recall that the noise model that we use is the global depolarizing noise channel E_λ_(ρ) = (1 − λ)ρ + λTr(ρ)*I_d_*/*d*, which acts independently on the subsystems of Alice and Bob, transforming a noiseless seed state σ into E_λ_ ⊗ E_λ_(σ). It turns out that, for a classical correlation *P*, if the strength of the quantum noise exceeds a certain threshold λ, then any quantum protocol will fail to generate *P*. Denote ${\left\|s\right\|}_{1}=\sum_{x=1}^{n}|s_{x}|$ as the one-norm of a vector **s** = (*s*_1_,…, *s_n_*). For a Hilbert space H, let *D*(H) denote the set of density operators on H. The following proposition can be used to identify this threshold. Note that, if $\sigma \overset{\lambda }{\to }P$ for some λ > 0, then every entry of *P* is positive. Thus, we always assume $P\in {\mathbb{R}}_{>0}^{n\times n}$ below.

**Proposition 1** Suppose $P\in {\mathbb{R}}_{>0}^{n\times n}$ is a correlation and λ < 1 is the noise strength. Then, there exists a dimension *d* and a quantum state σ ∈ *D*(ℂ*^d^* ⊗ ℂ*^d^*) such that $\sigma \overset{\lambda }{\to }P$, if and only if there exist $s=\left(s_{1},\ldots ,s_{n}\right),t=\left(t_{1},\ldots ,t_{n}\right)\in {\mathbb{R}}_{>0}^{n}$ with ‖**s**‖_1_ = ‖**t**‖_1_ = 1 such that

$${\hat{P}}_{\lambda }^{s,t}\left(x,y\right)\equiv P\left(x,y\right)-\lambda s_{x}\sum_{a}P\left(a,y\right)-\lambda t_{y}\sum_{b}P\left(x,b\right)+\lambda^{2}s_{x}t_{y}\geq 0$$ (4)

holds for all *x* and *y*.

The proof for Proposition 1 can be found in section S2. Here, we outline the main idea of the proof. If there exist POVMs {*E_x_*} and {*F_y_*} such that

$$\begin{array}{c} P\left(x,y\right)=\text{Tr}\left[E_{x}⊗F_{y}E_{\lambda }⊗E_{\lambda }\left(\sigma \right)\right]\\ ={\left(1-\lambda \right)}^{2}\text{Tr}\left(E_{x}⊗F_{y}\sigma \right)+\lambda \left(1-\lambda \right)\frac{\text{Tr}\left(E_{x}\right)}{d}\text{Tr}\left(F_{y}\sigma_{B}\right)\\ +\lambda \left(1-\lambda \right)\text{Tr}\left(E_{x}\sigma_{A}\right)\frac{\text{Tr}\left(F_{y}\right)}{d}+\lambda^{2}\frac{\text{Tr}\left(E_{x}\right)}{d}\frac{\text{Tr}\left(F_{y}\right)}{d} \end{array}$$ (5)

Take *s_x_* = Tr(*E_x_*)/*d* and *t_y_* = Tr(*F_y_*)/*d* and rearrange the terms. We then observe that ${\hat{P}}_{\lambda }^{s,t}\left(x,y\right)={\left(1-\lambda \right)}^{2}\text{Tr}\left(E_{x}⊗F_{y}\sigma \right)\geq 0$. Meanwhile, if Eq. 4 holds, we can first find a noiseless quantum protocol with a seed state σ′, and POVMs $\left\{E_{x}^{\prime }\right\}$ and $\left\{F_{y}^{\prime }\right\}$ to generate the correlation $\frac{1}{{\left(1-\lambda \right)}^{2}}{\hat{P}}_{\lambda }^{s,t}$. By enlarging the quantum systems of Alice and Bob to allow for more freedom, we can construct another quantum protocol that generates *P* exactly under the noise.

Now, we show how Proposition 1 helps to characterize the region of λ where a given classical correlation $P\in {\mathbb{R}}_{>0}^{n\times n}$ is reachable by quantum protocols. Let Λ(*P*) denote this region, i.e.

$$\Lambda \left(P\right)=\left\{\lambda :\text{there exists}\;a\;\text{quantum state}\;\rho \;\text{such that}\;\rho \overset{\lambda }{\to }P\right\}$$ (6)

For any λ ∈ Λ(*P*), according to Proposition 1, there exist $s,t\in {\mathbb{R}}_{>0}^{n}$ with ‖**s**‖_1_ = ‖**t**‖_1_ = 1, such that ${\hat{P}}_{\lambda }^{s,t}$ defined in Eq. 4, is a nonnegative matrix. Note that

$$\begin{array}{cc} \frac{\partial {\hat{P}}_{\lambda }^{s,t}\left(x,y\right)}{\partial \lambda } & =2\lambda s_{x}t_{y}-s_{x}\sum_{a}P\left(a,y\right)-t_{y}\sum_{b}P\left(x,b\right)\\ & =-\frac{1}{1-\lambda }\left[s_{x}\sum_{a}{\hat{P}}_{\lambda }^{s,t}\left(a,y\right)+t_{y}\sum_{b}{\hat{P}}_{\lambda }^{s,t}\left(x,b\right)\right]\leq 0 \end{array}$$ (7)

Thus, for any 0 ≤ λ′ ≤ λ we have ${\hat{P}}_{\lambda^{\prime }}^{s,t}\left(x,y\right)\geq {\hat{P}}_{\lambda }^{s,t}\left(x,y\right)\geq 0$, which implies that λ′ ∈ Λ(*P*). Therefore, Λ(*P*) is an interval. This indicates that, as λ increases continuously, the reachability of quantum protocols for *P* diminishes and will not be restored.

Proposition 1 fully characterizes the reachability of noisy quantum protocols in generating correlations. However, for a given *P*, determining whether suitable **s** and **t** can be found to satisfy the conditions in Proposition 1 is very challenging, making it difficult to ascertain whether a correlated *P* can be generated quantumly under the noise strength λ. To address this difficulty, we provide an easy-to-handle necessary condition for the reachability of noisy quantum protocols. Specifically, for a given classical correlation $P\in {\mathbb{R}}_{\geq 0}^{n\times n}$, a quantum state σ ∈ *D*(ℂ*^d^* ⊗ ℂ*^d^*) can satisfy $\sigma \overset{\lambda }{\to }P$ only if

$$0\leq \lambda \leq 1-\underset{\varphi \in S_{n}}{\text{max}}\sum_{x=1}^{n}\sqrt{\text{max}\left\{0,\sum_{b=1}^{n}P\left(x,b\right)\sum_{a=1}^{n}P\left(a,\varphi \left(x\right)\right)-P\left(x,\varphi \left(x\right)\right)\right\}}$$ (8)

where *S_n_* is the symmetric group of degree *n*. The proof is provided in section S3.

Note that $\sum_{\text{xy}}\left[\sum_{b}P(x,b)\sum_{a}P(a,y)-P(x,y)\right]=0$. Therefore, if rank(*P*) ≥ 2, then there will always be some *x*_0_ and *y*_0_ such that

$$\sum_{b}P\left(x_{0},b\right)\sum_{a}P\left(a,y_{0}\right)-P\left(x_{0},y_{0}\right)>0$$ (9)

Thus, the upper bound in Eq. 8 is always strictly less than 1. This means that, for any *P* with rank(*P*) ≥ 2, reachability will always be destroyed before λ reaches 1.

### Case of weak noise: Costs of noisy quantum protocols

For a given nontrivial classical correlation $P\in {\mathbb{R}}_{\geq 0}^{n\times n}$ with rank ≥ 2, we now focus on the interval Λ(*P*), the region of λ where *P* is reachable for noisy quantum protocols. In this context, an important problem is how the cost of generating *P* increases as the noise strength grows.

We denote C_λ_(*P*) as the minimum local dimension for the seed states that can generate *P* under the noise channel E_λ_ ⊗ E_λ_, i.e., the smallest number *d* such that there exists σ ∈ *D*(ℂ*^d^* ⊗ ℂ*^d^*) satisfying $\sigma \overset{\lambda }{\to }P$. Thus, the size of the minimum seed state can be written as ⌈log_2_C_λ_(*P*)⌉.

Recall that the cost of the optimal noiseless quantum protocol generating *P* can be fully characterized as

$$Q\left(P\right)=\left\lceil {\text{log}}_{2}{\text{rank}}_{\text{psd}}\left(P\right)\right\rceil$$ (10)

where rank_psd_(*P*) is the PSD rank of *P* (*45*). Inspired by this result, we connect the cost of noisy quantum protocols in generating correlations to a modified version of PSD rank. Specifically, we define ${\text{rank}}_{\text{psd}}^{E_{\lambda }}\left(P\right)$ as the smallest *r* for which there exist *r* × *r* PSD matrices {*C_x_*}, {*D_y_*} such that *P*(*x*, *y*) = Tr(*C_x_D_y_*), with both

$$C_{x}-\frac{\lambda }{r}\text{Tr}\left[C_{x}{\left(\sum_{k=1}^{n}C_{k}\right)}^{-1}\right]\sum_{k=1}^{n}C_{k}$$ (11)

and

$$D_{y}-\frac{\lambda }{r}\text{Tr}\left[D_{y}{\left(\sum_{k=1}^{n}D_{k}\right)}^{-1}\right]\sum_{k=1}^{n}D_{k}$$ (12)

are PSD matrices. In this context, we say {*C_x_*}, {*D_y_*} is an E_λ_-PSD factorization of *P*. Then, we have that

$$C_{\lambda }\left(P\right)\leq {\text{rank}}_{\text{psd}}^{E_{\lambda }}\left(P\right)$$ (13)

which will be proven in section S4. In particular, if we restrict seed states to be pure states, then the above inequality holds with equality.

Meanwhile, Proposition 1 helps in bounding C_λ_(*P*), allowing us to obtain the following upper and lower bounds for C_λ_(*P*). For a given classical correlation $P\in {\mathbb{R}}_{>0}^{n\times n}$ and λ ≥ 0, if there exist $s,t\in {\mathbb{R}}_{>0}^{n}$ with ‖**s**‖_1_ = ‖**t**‖_1_ = 1 such that ${\hat{P}}_{\lambda }^{s,t}\left(x,y\right)$ defined in Eq. 4 is nonnegative for all *x* and *y*, then

$$C_{\lambda }\left(P\right)\leq {\text{rank}}_{\text{psd}}\left({\hat{P}}_{\lambda }^{s,t}\right)\left\lceil \frac{1}{\underset{x,y}{\text{min}}\left\{s_{x},t_{y}\right\}}\right\rceil$$ (14)

and

$$C_{\lambda }\left(P\right)\geq \frac{1}{1-\lambda }\left(\underset{s\prime ,t\prime }{\text{inf}}\;\underset{x,y}{\text{max}}\left\{\frac{\sum_{b}P\left(x,b\right)}{s\prime_{x}},\frac{\sum_{a}P\left(a,y\right)}{t\prime_{y}}\right\}-\lambda \right)$$ (15)

where $s\prime ,t\prime \in {\mathbb{R}}_{>0}^{n}$ with ‖**s**′‖_1_ = ‖**t**′‖_1_ = 1 satisfy that ${\hat{P}}_{\lambda }^{s^{\prime },t^{\prime }}\left(x,y\right)\geq 0$. The proofs for these bounds can be found in section S5.

### Decay process of quantum advantage

Now, we are ready to analyze how quantum advantage is affected as the noise strength increases continuously. We will demonstrate our techniques using specific examples.

**Fig. 1.**
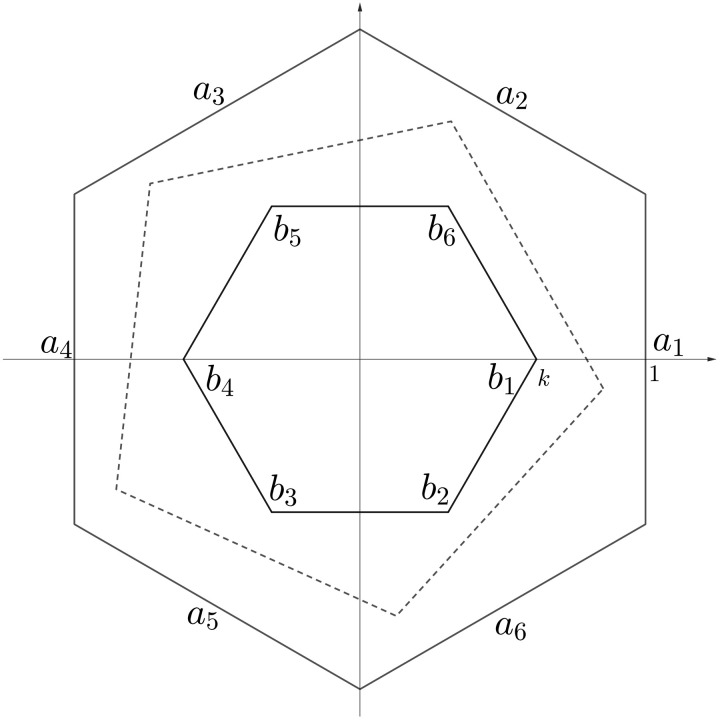
Geometric interpretation for *B_m_*, where *m* = 6. Vertices of smaller solid-line polygon are labeled clockwise from 1 to 6, while edges of bigger solid-line polygon are labeled counterclockwise from 1 to 6. Dash-line pentagon shows geometric interpretation for restricted nonnegative rank of *B_m_*.

Define $A_{m}\in {\mathbb{R}}^{\left(m+1)\times (m+1\right)}$ as the correlation with entries given by

$$A_{m}=\left(\begin{array}{cccc} \frac{{\left(1-q\right)}^{2}}{2} & \frac{q\left(1-q\right)}{2m} & \cdots & \frac{q\left(1-q\right)}{2m}\\ \frac{q\left(1-q\right)}{2m} & & & \\ \vdots & & \frac{1+q^{2}}{2}B_{m} & \\ \frac{q\left(1-q\right)}{2m} & & & \end{array}\right)$$ (16)

where $q=\frac{1}{1-k}-\sqrt{\frac{1}{{\left(1-k\right)}^{2}}-1}$, *k* is a parameter, and *B_m_* is an *m* × *m* correlation with entries given by

$$B_{m}\left(x,y\right)=\frac{1}{m^{2}}\left[1-k\;\text{cos}\left(2\pi \frac{x-1+y-1}{m}\right)\right]$$ (17)

for *x*, *y* ∈ [*m*]. Note that, if 0 < *k* < 1, then we have 0 < *q* < 1. The correlation *B_m_*, as a matrix, is the slack matrix of two concentric regular polygons, as shown in (Fig. 1). More details about this geometric interpretation can be found in (*47*, *52*), and additional mathematical properties of *A_m_* and *B_m_* are provided in section S6.

It can be shown that, in the absence of noise, *A_m_* exhibits an arbitrarily large quantum advantage as *m* → ∞. As we prove in section S6, if $k>\frac{\text{cos}\left(2\pi /m\right)}{{\text{cos}}^{2}\left(\pi /m\right)}$, then rank_+_(*A_m_*) > log_2_(*m*/2), whereas rank_psd_(*A_m_*) ≤ 3. As a result, this indicates that

$$\underset{m\to \infty }{\text{lim}}S_{0}\left(A_{m}\right)=\underset{m\to \infty }{\text{lim}}\frac{R\left(A_{m}\right)}{\left\lceil {\text{log}}_{2}{\text{rank}}_{\text{psd}}\left(A_{m}\right)\right\rceil }=\infty$$ (18)

We now turn to noisy quantum protocols that generate *A_m_*. Recall that ⌈log_2_C_λ_(*A_m_*)⌉ is the smallest size of a seed state that can generate *A_m_* under the noise channel E_λ_ ⊗ E_λ_, which can be upper and lower bounded by Eqs. 14 and 15. As a result, we can characterize the decay of quantum advantage in generating *A_m_* as λ increases.

**Theorem 2** As the noise strength λ approaches *q*, the quantum advantage in generating *A_m_* asymptotically decreased to 0.

The proof for Theorem 2 can be found in section S7. Here, we sketch the main idea of the proof. Let λ = *q* − ϵ, where 0 < ϵ < *q*.

First, it can be verified that, for any 0 < ϵ < *q*, *A_m_* remains reachable. To show this, we can choose suitable **s**, **t** to satisfy Eq. 4 with *P* = *A_m_*. According to Proposition 1, *A_m_* is reachable when 0 < ϵ < *q*. In addition, using the upper bound in Eq. 14, the minimum local dimension for the seed states C_λ_(*A_m_*) can be bounded as $O\left(\epsilon^{-1}\right)$.

Second, as ϵ → 0, using the lower bound in Eq. 15 and the fact that $\text{min}\left\{s_{1},t_{1}\right\}=O\left(\epsilon^{1/2}\right)\to 0$, we find that C_λ_(*A_m_*) = Ω(ϵ^−1/2^) → ∞.

In conclusion, we have

$$S_{\lambda }\left(A_{m}\right)=\frac{R\left(A_{m}\right)}{\left\lceil {\text{log}}_{2}C_{\lambda }\left(A_{m}\right)\right\rceil }=\Theta \left(\frac{1}{\text{log}\epsilon^{-1}}\right)$$ (19)

where we have used the fact that classical protocols are noiseless, meaning that R(*A_m_*) is independent of ϵ. In other words, as λ → *q*, C_λ_(*A_m_*) → ∞, implying that $S_{\lambda }\left(A_{m}\right)\to 0$. This demonstrates that, as λ approaches *q*, although *A_m_* remains reachable for noisy quantum protocols, the costs will increase without bound, ultimately destroying the quantum advantage that noiseless protocols would provide in generating *A_m_*.

### Sudden death of quantum advantage

We have shown that $S_{\lambda }\left(A_{m}\right)$ decays asymptotically to 0 as the noise rate λ increases. Unexpectedly, if we instead focus on *B_m_*, a subcorrelation of *A_m_*, then an unexpected phenomenon occurs: As λ continuously increases beyond a certain point, $S_{\lambda }\left(B_{m}\right)$ suddenly drops to 0 from a non-negligible value. We refer to this as the sudden death of quantum advantage in correlation generation.

**Theorem 3** Let 0 < *k* < 1. For any integer *m* and the classical correlation *B_m_* defined in Eq. 16, we have

1) $S_{\lambda }\left(B_{m}\right)=\Omega \left(\text{loglog}\left(m\right)\right)$, if $0\leq \lambda \leq 1-\sqrt{k}$;

2) $S_{\lambda }\left(B_{m}\right)=0$, if $\lambda >1-\sqrt{k}$.

Therefore, the quantum advantage in generating *B_m_* experiences sudden death when the noise strength exceeds $\lambda =1-\sqrt{k}$.

Proof: Let φ(1) = 1, φ(*x*) = *m* + 2 − *x*. We have *B_m_*[*x*, φ(*x*)] = (1 − *k*)/*m*^2^ for all *x*. Then

$$\sum_{b}B_{m}\left(x,b\right)\sum_{a}B_{m}\left(a,\varphi \left(x\right)\right)-B_{m}\left(x,\varphi \left(x\right)\right)=\frac{k}{m^{2}}$$ (20)

By Eq. 8, if *B_m_* can be generated under noise E_λ_ ⊗ E_λ_, then we must have

$$0\leq \lambda \leq 1-\sum_{x=1}^{m}\sqrt{\text{max}\left\{0,\sum_{b}B_{m}\left(x,b\right)\sum_{a}B_{m}\left(a,\varphi \left(x\right)\right)-B_{m}\left(x,\varphi \left(x\right)\right)\right\}}=1-\sqrt{k}$$ (21)

Thus, for $\lambda >1-\sqrt{k}$, *B_m_* is not reachable by any noisy quantum protocols, meaning that $S_{\lambda }\left(B_{m}\right)=\frac{R\left(B_{m}\right)}{\infty }=0$.

For $0\leq \lambda \leq 1-\sqrt{k}$, we select the 2 × 2 Hermitian matrices {*C_x_*} and {*D_y_*} introduced in Lemma S1 (section S6) as a PSD decomposition of *B_m_*, and we have

$$\begin{array}{c} C_{x}-\frac{\lambda }{r}\text{Tr}\left[C_{x}{\left(\sum_{k=1}^{n}C_{k}\right)}^{-1}\right]\sum_{k=1}^{n}C_{k}=C_{x}-\frac{\lambda }{\sqrt{2}m}I\\ D_{y}-\frac{\lambda }{r}\text{Tr}\left[D_{y}{\left(\sum_{k=1}^{n}D_{k}\right)}^{-1}\right]\sum_{k=1}^{n}D_{k}=D_{y}-\frac{\lambda }{\sqrt{2}m}I \end{array}$$ (22)

The minimum eigenvalues of *C_x_* and *D_y_* are $\frac{1}{\sqrt{2}m}\left(1-\sqrt{k}\right)$, meaning that both $C_{i}-\frac{\lambda }{\sqrt{2}m}I$ and $D_{j}-\frac{\lambda }{\sqrt{2}m}I$ are PSD matrices. Thus, {*C_x_*} and {*D_y_*} form an E_λ_-PSD factorization of *B_m_*. By Eq. 13, we have

$$C_{\lambda }\left(B_{m}\right)\leq {\text{rank}}_{\text{psd}}^{E_{\lambda }}\left(B_{m}\right)\leq 2$$ (23)

Because *B_m_* is not a product distribution, we have C_λ_(*B_m_*) = 2. Therefore, for $0\leq \lambda \leq 1-\sqrt{k}$, we have

$$S_{\lambda }\left(B_{m}\right)=\frac{R\left(B_{m}\right)}{\left\lceil {\text{log}}_{2}C_{\lambda }\left(B_{m}\right)\right\rceil }=R\left(B_{m}\right)$$ (24)

which is a constant independent of λ. By Lemma 1 (section S6), R(*B_m_*) ≥ ⌈log_2_log_2_(*m*/2)⌉, which completes the proof.

It is possible to observe the sudden death of quantum advantage experimentally. When $\lambda >1-\sqrt{k}$, we have shown that *B_m_* cannot be generated by any quantum system in our setting. However, when $0\leq \lambda \leq 1-\sqrt{k}$, as demonstrated in the proof of Theorem 3, *B_m_* can always be generated by a qubit-qubit system, which can be physically demonstrated by many quantum experimental platforms, as the required system size is constant and small.

For any classical correlation *P*, when the sudden death of quantum advantage occurs, $S_{\lambda }\left(P\right)$ will be lower bounded by a positive constant independent of λ for any λ ∈ Λ(*P*). This implies that sup_λ∈Λ(*P*)_C_λ_(*P*) < ∞. To figure out when such a phenomenon occurs, we provide two equivalent conditions below.

**Theorem 4** For any correlation $P\in {\mathbb{R}}_{>0}^{n\times n}$, the following statements are equivalent:

1) The quantum advantage in generating *P* experiences sudden death as λ increases from 0.

2) Λ(*P*) is a right-closed interval.

3) For any λ > 0, if there exist nonnegative vectors $s,t\in {\mathbb{R}}_{\geq 0}^{n}$ with ‖**s**‖_1_ = ‖**t**‖_1_ = 1 such that ${\hat{P}}_{\lambda }^{s,t}$ defined in Eq. 4 is a nonnegative matrix, then *P* can be generated quantumly under the noise channel E_λ_ ⊗ E_λ_.

The proof for Theorem 4 is deferred to section S8. As a simple application of this theorem, we can reexamine the correlation *B_m_*. According to the proof of Theorem 3, we have $\Lambda \left(B_{m}\right)=\left[0,1-\sqrt{k}\right]$, which is right closed. Then, Theorem 4 directly implies that the quantum advantage in generating *B_m_* suffers from sudden death.

## DISCUSSION

On the basis of an elegant sampling model where quantum advantage has been both identified and quantified, we mathematically characterize the dynamical process by which increasing noise gradually suppresses and eventually destroys quantum advantage. In addition, we report the unexpected phenomenon of sudden death of quantum advantage under the impact of noise in this model. The sudden death of quantum advantage demonstrated by our quantum protocol implies that the impact of noise may exhibit different behaviors across various quantum information processing tasks. Our work suggests that, to fully understand the potential of NISQ computers, further efforts are needed to characterize the nature of quantum noise across different computational models, especially the quantum circuit model.

## Supplementary Materials

### This PDF file includes:

Sections S1 to S8 References
