# Supplementary material for: Sudden death of quantum advantage in correlation generations
Source: Sci Adv. 2024 Nov 22;10(47):eadr5002. doi: 10.1126/sciadv.adr5002 (PMC13108740; doi:10.1126/sciadv.adr5002)
Supplement: Supplementary file 1 — Sections S1 to S8 References [file sciadv.adr5002_sm.pdf]

Science Advances
AAAS

Supplementary Materials for

Sudden death of quantum advantage in correlation generations

Weixiao Sun *et al.*

Corresponding author: Zhaohui Wei, weizhaohui@gmail.com

Sci. Adv. **10**, eadr5002 (2024)
DOI: 10.1126/sciadv.adr5002

This PDF file includes:

Sections S1 to S8
References

Section S1. Proof for Theorem 1

Theorem S1 *Suppose $\rho = |\psi\rangle\langle\psi|$ is a bipartite entangled pure quantum state on $\mathcal{H}_A \otimes \mathcal{H}_B$. Then, there always exists a family of classical correlations $\{P_m\}_{m\in\mathbb{Z}^+}$ such that $\rho \xrightarrow{0} P_m$ for all m, and $\lim_{m\to\infty} \mathcal{S}_0(P_m) = \infty$.*

Proof: Suppose $\dim(\mathcal{H}_A) = \dim(\mathcal{H}_B) = d \geq 2$, the Schmidt decomposition of $|\psi\rangle$ is

$$|\psi\rangle = \sum_{i=1}^{d} \sqrt{\lambda_i}|\phi_i^A\rangle \otimes |\phi_i^B\rangle, \tag{S1}$$

where $\sqrt{\lambda_1} \geq \cdots \geq \sqrt{\lambda_d} > 0$ are the non-zero Schmidt coefficients. Denote $\mu_1 = \frac{\lambda_1}{\lambda_1+\lambda_2}$, $\mu_2 = \frac{\lambda_2}{\lambda_1+\lambda_2}$, and let

$$|\psi'\rangle = \sqrt{\mu_1}|\phi_1^A\rangle \otimes |\phi_1^B\rangle + \sqrt{\mu_2}|\phi_2^A\rangle \otimes |\phi_2^B\rangle. \tag{S2}$$

Take $\Pi^A = |\phi_1^A\rangle\langle\phi_1^A| + |\phi_2^A\rangle\langle\phi_2^A|$, $\Pi^B = |\phi_1^B\rangle\langle\phi_1^B| + |\phi_2^B\rangle\langle\phi_2^B|$ be two projectors; we have $\Pi^A \otimes \Pi^B|\psi\rangle = \sqrt{\lambda_1 + \lambda_2}|\psi'\rangle$.

For m distinct real numbers $\alpha_1, \cdots, \alpha_m$, define the $m \times m$ Euclidean distance matrix EDM_m by $\mathrm{EDM}_m(x, y) = \frac{(\alpha_x-\alpha_y)^2}{\sum_{ij}(\alpha_i-\alpha_j)^2}$. For arbitrary m, we can pick $\alpha_1, \cdots, \alpha_m$ as described in (*51*), such that

1. $\mathrm{rank}_{\mathrm{psd}}(\mathrm{EDM}_m) = 2$ and $\mathrm{rank}_+(\mathrm{EDM}_m) \geq 2\sqrt{m} - 2$,
2. $\alpha_1 + \cdots + \alpha_m = 0$,
3. α_1 big enough, such that $\sum_{y=1}^m \mathrm{EDM}_m(1, y) = \frac{\sum_y(\alpha_1-\alpha_y)^2}{\sum_{ij}(\alpha_i-\alpha_j)^2} > \mu_1 - \frac{1}{2}$.

For $x, y = 1, \cdots, m$, let

$$C_x = \frac{1}{\sqrt{2}}\begin{pmatrix} \frac{\alpha_x^2}{\sum_i \alpha_i^2} & \frac{-\alpha_x}{\sqrt{m\sum_i \alpha_i^2}} \\ \frac{-\alpha_x}{\sqrt{m\sum_i \alpha_i^2}} & \frac{1}{m} \end{pmatrix},$$
$$D_y = \frac{1}{\sqrt{2}}\begin{pmatrix} \frac{1}{m} & \frac{\alpha_y}{\sqrt{m\sum_i \alpha_i^2}} \\ \frac{\alpha_y}{\sqrt{m\sum_i \alpha_i^2}} & \frac{\alpha_y^2}{\sum_i \alpha_i^2} \end{pmatrix}. \tag{S3}$$

By $\sum_i \alpha_i = 0$, we know that $\sum_{ij}(\alpha_i - \alpha_j)^2 = 2m\sum_i \alpha_i^2$, thus we have $\mathrm{Tr}\left(C_x D_y\right) = \frac{(\alpha_x-\alpha_y)^2}{2m\sum_i \alpha_i^2} = \mathrm{EDM}_m(x, y)$. Meanwhile, it can be verified that

$$\sum_x C_x = \sum_y D_y = \begin{pmatrix} \frac{1}{\sqrt{2}} & \\ & \frac{1}{\sqrt{2}} \end{pmatrix}. \tag{S4}$$

Denote $r = \frac{\mu_1 - 1/2}{\sum_{y=1}^{m} \mathrm{EDM}_m(1,y)}$, then we have $0 \le r < 1$. Let $\mathrm{EDM}'_m = L \cdot \mathrm{EDM}_m \cdot R$, where

$$\begin{aligned} L &= \mathrm{diag}\,(1+r, 1, \cdots, 1)\,, \\ R &= \mathrm{diag}\,(1-r, 1, \cdots, 1)\,. \end{aligned} \tag{S5}$$

Since L and R are diagonal matrices with diagonal elements > 0, we have $\mathrm{rank}_+(\mathrm{EDM}'_m) = \mathrm{rank}_+(\mathrm{EDM}_m)$. Note that

$$\begin{aligned} &\{(1+r)C_1, C_2, \cdots, C_m\}, \\ &\{(1-r)D_1, D_2, \cdots, D_m\} \end{aligned} \tag{S6}$$

form a PSD decomposition of EDM'_m. Since $C_1, D_1 \in \mathbb{C}^{2\times 2}$ are PSD matrices satisfying $\mathrm{Tr}\,(C_1 D_1) = \mathrm{EDM}_m(1,1) = 0$, we know that C_1 and D_1 are two rank-1 projectors with $C_1 D_1 = 0$. Thus, the eigenvalues of

$$\begin{aligned} \left((1+r)C_1 + \sum_{x=2}^{m} C_x\right)\left((1-r)D_1 + \sum_{y=2}^{m} D_y\right) &= \left(rC_1 + \frac{1}{\sqrt{2}}I_2\right)\left(-rD_1 + \frac{1}{\sqrt{2}}I_2\right) \\ &= \frac{1}{2}I_2 + \frac{r}{\sqrt{2}}(C_1 - D_1) \end{aligned} \tag{S7}$$

are

$$\begin{aligned} \frac{1}{2} + \frac{r}{\sqrt{2}}\mathrm{Tr}\,(C_1) &= \frac{1}{2} + \frac{r}{\sum_{y=1}^{m} \mathrm{EDM}_m(1,y)} = \mu_1, \\ \frac{1}{2} - \frac{r}{\sqrt{2}}\mathrm{Tr}\,(D_1) &= \frac{1}{2} - \frac{r}{\sum_{y=1}^{m} \mathrm{EDM}_m(1,y)} = \mu_2. \end{aligned} \tag{S8}$$

Denote the diagonal form of the PSD factorization of EDM'_m equivalent to Eq.(S6) by $\{C'_x, D'_y\}$, where by equivalence we mean that there exists an invertible H such that $C'_x = HC_xH^\dagger$ and $D'_y = (H^\dagger)^{-1} D_y H^{-1}$ (such an H always exists (*53*)). Then we have $\mathrm{Tr}\left(C'_x D'_y\right) = \mathrm{EDM}'_m(x,y)$ and

$$\sum_x C'_x = \sum_y D'_y = \begin{pmatrix} \sqrt{\mu_1} & \\ & \sqrt{\mu_2} \end{pmatrix}, \tag{S9}$$

which coincides with the Schmidt coefficients of $|\psi'\rangle$. By Theorem 2 of (*54*), there exist POVMs $\{E_x\}$ and $\{F_y\}$ on $\Pi^A\mathcal{H}_A \otimes \Pi^B\mathcal{H}_B$ (i.e., $\Pi^A E_x \Pi^A = E_x$, $\Pi^B F_y \Pi^B = F_y$, $\sum_x E_x = \Pi^A$, and $\sum_y F_y = \Pi^B$) such that $\mathrm{Tr}\left(E_x \otimes F_y |\psi'\rangle\langle\psi'|\right) = \mathrm{EDM}'_m(x,y)$.

Now consider measuring POVMs

$$\begin{aligned} &\{E_1, \cdots, E_m, I - \Pi^A\}, \\ &\{F_1, \cdots, F_m, I - \Pi^B\}, \end{aligned} \tag{S10}$$

on the two subsystems of $|\psi\rangle\langle\psi|$ respectively. Since

$$\begin{aligned}
&\mathrm{Tr}\left(E_x \otimes F_y |\psi\rangle\langle\psi|\right) = (\lambda_1 + \lambda_2)\mathrm{EDM}'_m(x, y),\\
&\mathrm{Tr}\left(E_x \otimes (I - \Pi^B)|\psi\rangle\langle\psi|\right) = 0,\\
&\mathrm{Tr}\left((I - \Pi^A) \otimes F_y|\psi\rangle\langle\psi|\right) = 0,\\
&\mathrm{Tr}\left((I - \Pi^A) \otimes (I - \Pi^B)|\psi\rangle\langle\psi|\right) = 1 - \lambda_1 - \lambda_2,
\end{aligned} \tag{S11}$$

we have generated an $(m + 1) \times (m + 1)$ correlation

$$P_m = \begin{pmatrix} (\lambda_1 + \lambda_2)\mathrm{EDM}'_m & 0 \\ 0 & 1 - \lambda_1 - \lambda_2 \end{pmatrix} \tag{S12}$$

with the seed state $\rho = |\psi\rangle\langle\psi|$. And it holds that

$$\mathrm{rank}_+(P_m) \geq \mathrm{rank}_+(\mathrm{EDM}'_m) = \mathrm{rank}_+(\mathrm{EDM}_m) \geq 2\sqrt{m} - 2. \tag{S13}$$

Since m can be arbitrarily large, we have $\lim_{m\to\infty} \mathcal{S}_0(P_m) = \infty$.

Section S2. Proof for Proposition 1

Proposition S1 *Suppose $P \in \mathbb{R}^{n\times n}_{>0}$ is a correlation and $\lambda < 1$ is a noise strength. Then there exist a dimension d and a quantum state $\sigma \in D(\mathbb{C}^d \otimes \mathbb{C}^d)$ making $\sigma \xrightarrow{\lambda} P$, if and only if there exist $\mathbf{s}, \mathbf{t} \in \mathbb{R}^n_{>0}$ with $\|\mathbf{s}\|_1 = \|\mathbf{t}\|_1 = 1$ such that*

$$\hat{P}^{\mathbf{s},\mathbf{t}}_\lambda(x, y) \equiv P(x, y) - \lambda s_x \sum_a P(a, y) - \lambda t_y \sum_b P(x, b) + \lambda^2 s_x t_y \geq 0 \tag{S14}$$

holds for all x, y.

Proof: ($\Rightarrow$) Note that

$$
\begin{aligned}
P(x,y) =& \mathrm{Tr}\left(E_x \otimes F_y \mathcal{E}_\lambda \otimes \mathcal{E}_\lambda(\sigma)\right) \\
=& (1-\lambda)^2 \mathrm{Tr}\left(E_x \otimes F_y \sigma\right) + \lambda(1-\lambda)\frac{\mathrm{Tr}\left(E_x\right)}{d}\mathrm{Tr}\left(F_y \sigma_B\right) \\
& + \lambda(1-\lambda)\mathrm{Tr}\left(E_x \sigma_A\right)\frac{\mathrm{Tr}\left(F_y\right)}{d} + \lambda^2 \frac{\mathrm{Tr}\left(E_x\right)}{d}\frac{\mathrm{Tr}\left(F_y\right)}{d} \\
=& (1-\lambda)^2 \mathrm{Tr}\left(E_x \otimes F_y \sigma\right) + \lambda \frac{\mathrm{Tr}\left(E_x\right)}{d}\left(\sum_a P(a,y) - \lambda \frac{\mathrm{Tr}\left(F_y\right)}{d}\right) \\
& + \lambda \frac{\mathrm{Tr}\left(F_y\right)}{d}\left(\sum_b P(x,b) - \lambda \frac{\mathrm{Tr}\left(E_x\right)}{d}\right) + \lambda^2 \frac{\mathrm{Tr}\left(E_x\right)}{d}\frac{\mathrm{Tr}\left(F_y\right)}{d} \\
=& (1-\lambda)^2 \mathrm{Tr}\left(E_x \otimes F_y \sigma\right) + \lambda \frac{\mathrm{Tr}\left(E_x\right)}{d}\sum_a P(a,y) \\
& + \lambda \frac{\mathrm{Tr}\left(F_y\right)}{d}\sum_b P(x,b) - \lambda^2 \frac{\mathrm{Tr}\left(E_x\right)}{d}\frac{\mathrm{Tr}\left(F_y\right)}{d},
\end{aligned}
\tag{S15}
$$

where $\sigma_A = \mathrm{Tr}_B(\sigma)$ and $\sigma_B = \mathrm{Tr}_A(\sigma)$ are the reduced density matrices. If we can take $s_x = \frac{\mathrm{Tr}(E_x)}{d}$, $t_y = \frac{\mathrm{Tr}(F_y)}{d}$, then

$$
P(x,y) - \lambda s_x \sum_a P(a,y) - \lambda t_y \sum_b P(x,b) + \lambda^2 s_x t_y = (1-\lambda)^2 \mathrm{Tr}\left(E_x \otimes F_y \sigma\right) \geq 0. \tag{S16}
$$

With the properties of POVM, we have $\mathbf{s}, \mathbf{t} \in \mathbb{R}^n_{>0}$, and $\|\mathbf{s}\|_1 = \sum_x \frac{\mathrm{Tr}(E_x)}{d} = \frac{\mathrm{Tr}(I_d)}{d} = 1$. Similarly, it holds that $\|\mathbf{t}\|_1 = 1$.

($\Leftarrow$) Denote that

$$
\hat{P}^{\mathbf{s},\mathbf{t}}_\lambda(x,y) = P(x,y) - \lambda s_x \sum_a P(a,y) - \lambda t_y \sum_b P(x,b) + \lambda^2 s_x t_y, \tag{S17}
$$

which naturally satisfies $\sum_{xy} \frac{1}{(1-\lambda)^2}\hat{P}^{\mathbf{s},\mathbf{t}}_\lambda(x,y) = 1$.

Hence, $\frac{1}{(1-\lambda)^2}\hat{P}^{\mathbf{s},\mathbf{t}}_\lambda$ can be regarded as a correlation. Based on the results in (*45*), we know that there exist POVMs $\{E'_x\}$ and $\{F'_y\}$, and a quantum state $\sigma' \in D(\mathbb{C}^{d'} \otimes \mathbb{C}^{d'})$ such that $\mathrm{Tr}\left(E'_x \otimes F'_y \sigma'\right) = \frac{1}{(1-\lambda)^2}\hat{P}^{\mathbf{s},\mathbf{t}}_\lambda(x,y)$, where $d' = \mathrm{rank}_{\mathrm{psd}}(\hat{P}^{\mathbf{s},\mathbf{t}}_\lambda)$.

Take $k \in \mathbb{Z}^+$ such that

$$
\begin{aligned}
d' k s_x - \mathrm{Tr}\left(E'_x\right) \geq 0, \\
d' k t_y - \mathrm{Tr}\left(F'_y\right) \geq 0,
\end{aligned}
\tag{S18}
$$

for all x, y. Denote $d = d'k$ and $\sigma = |0\rangle\langle 0| \otimes \sigma' \otimes |0\rangle\langle 0|$, where $|0\rangle\langle 0| \in D(\mathbb{C}^k)$. Let

$$\begin{aligned} E_x &= |0\rangle\langle 0| \otimes E'_x + \left(\frac{d'ks_x - \mathrm{Tr}\left(E'_x\right)}{k-1}\right)(I_k - |0\rangle\langle 0|) \otimes \frac{I_{d'}}{d'}, \\ F_y &= F'_y \otimes |0\rangle\langle 0| + \left(\frac{d'kt_y - \mathrm{Tr}\left(F'_y\right)}{k-1}\right)\frac{I_{d'}}{d'} \otimes (I_k - |0\rangle\langle 0|), \end{aligned} \tag{S19}$$

which are PSD matrices. Note that

$$\begin{aligned} \sum_x E_x &= |0\rangle\langle 0| \otimes \sum_x E'_x + \left(\frac{d'k\sum_x s_x - \mathrm{Tr}\left(\sum_x E'_x\right)}{k-1}\right)(I_k - |0\rangle\langle 0|) \otimes \frac{I_{d'}}{d'} \\ &= |0\rangle\langle 0| \otimes I_{d'} + \left(\frac{d'k - d'}{k-1}\right)(I_k - |0\rangle\langle 0|) \otimes \frac{I_{d'}}{d'} = I_k \otimes I_{d'} = I_d, \\ \sum_y F_y &= \sum_y F'_y \otimes |0\rangle\langle 0| + \left(\frac{d'k\sum_b t_b - \mathrm{Tr}\left(\sum_y F'_y\right)}{k-1}\right)\frac{I_{d'}}{d'} \otimes (I_k - |0\rangle\langle 0|) \\ &= I_{d'} \otimes |0\rangle\langle 0| + \left(\frac{d'k - d'}{k-1}\right)\frac{I_{d'}}{d'} \otimes (I_k - |0\rangle\langle 0|) = I_{d'} \otimes I_k = I_d, \end{aligned} \tag{S20}$$

thus $\{E_x\}$, $\{F_y\}$ are valid POVMs. We also have

$$\begin{aligned} \mathrm{Tr}\left(E_x \otimes F_y \sigma\right) &= \mathrm{Tr}\Big(|0\rangle\langle 0| \otimes E'_x \otimes F'_y \otimes |0\rangle\langle 0| \cdot |0\rangle\langle 0| \otimes \sigma' \otimes |0\rangle\langle 0| + 0 + 0 + 0\Big) \\ &= \frac{1}{(1-\lambda)^2}\hat{P}^{\mathbf{s},\mathbf{t}}_{\lambda}(x, y), \\ \mathrm{Tr}\left(E_x\right) &= \mathrm{Tr}\left(E'_x\right) + \left(\frac{d'ks_x - \mathrm{Tr}\left(E'_x\right)}{k-1}\right)(k-1) = d'ks_x = ds_x, \\ \mathrm{Tr}\left(F_y\right) &= \mathrm{Tr}\left(F'_y\right) + \left(\frac{d'kt_y - \mathrm{Tr}\left(F'_y\right)}{k-1}\right)(k-1) = d'kt_y = dt_y. \end{aligned} \tag{S21}$$

Finally, note that

$$\begin{aligned} \sum_a \hat{P}^{\mathbf{s},\mathbf{t}}_{\lambda}(a, y) &= (1-\lambda)\left(\sum_a P(a, y) - \lambda t_y\right), \\ \sum_b \hat{P}^{\mathbf{s},\mathbf{t}}_{\lambda}(x, b) &= (1-\lambda)\left(\sum_b P(x, b) - \lambda s_x\right). \end{aligned} \tag{S22}$$

Thus, we have

$$
\begin{aligned}
&\mathrm{Tr}\left(E_x \otimes F_y \mathcal{E}_\lambda \otimes \mathcal{E}_\lambda(\sigma)\right) \\
=&(1-\lambda)^2 \mathrm{Tr}\left(E_x \otimes F_y \sigma\right) + \lambda(1-\lambda)\frac{\mathrm{Tr}\left(E_x\right)}{d}\mathrm{Tr}\left(F_y \sigma_B\right) \\
&+ \lambda(1-\lambda)\mathrm{Tr}\left(E_x \sigma_A\right)\frac{\mathrm{Tr}\left(F_y\right)}{d} + \lambda^2 \frac{\mathrm{Tr}\left(E_x\right)}{d}\frac{\mathrm{Tr}\left(F_y\right)}{d} \\
=&\hat{P}_\lambda^{\mathbf{s},\mathbf{t}}(x,y) + \frac{\lambda}{1-\lambda}s_x\left(\sum_a \hat{P}_\lambda^{\mathbf{s},\mathbf{t}}(a,y)\right) + \frac{\lambda}{1-\lambda}t_y\left(\sum_b \hat{P}_\lambda^{\mathbf{s},\mathbf{t}}(x,b)\right) + \lambda^2 s_x t_y \\
=&\hat{P}_\lambda^{\mathbf{s},\mathbf{t}}(x,y) + \lambda s_x\left(\sum_a P(a,y) - \lambda t_y\right) + \lambda t_y\left(\sum_b P(x,b) - \lambda s_x\right) + \lambda^2 s_x t_y \\
=&P(x,y).
\end{aligned} \tag{S23}
$$

Section S3. Proof for Eq.(8)

We restate the conclusion in Eq.(8): For a given classical correlation $P \in \mathbb{R}_{\geq 0}^{n\times n}$, there exists a quantum state $\sigma \in D(\mathbb{C}^d \otimes \mathbb{C}^d)$ satisfying $\sigma \xrightarrow{\lambda} P$, only if

$$
0 \leq \lambda \leq 1 - \max_{\varphi \in S_n} \sum_{x=1}^{n} \sqrt{\max\left\{0, \sum_b P(x,b)\sum_a P(a,\varphi(x)) - P(x,\varphi(x))\right\}}, \tag{S24}
$$

where S_n is a symmetric group of degree n.

Proof: By Proposition 1, there exist $\mathbf{s},\mathbf{t} \in \mathbb{R}_{>0}^n$ with $\|\mathbf{s}\|_1 = \|\mathbf{t}\|_1 = 1$ satisfying that

$$
\hat{P}_\lambda^{\mathbf{s},\mathbf{t}}(a,b) = P(a,b) - \lambda s_a \sum_a P(a,b) - \lambda t_b \sum_b P(a,b) + \lambda^2 s_a t_b \geq 0. \tag{S25}
$$

Then, summing a and b separately yields

$$
\begin{aligned}
\sum_a P(a,y) &= \frac{1}{1-\lambda}\sum_a \hat{P}_\lambda^{\mathbf{s},\mathbf{t}}(a,y) + \lambda t_y, \\
\sum_b P(x,b) &= \frac{1}{1-\lambda}\sum_b \hat{P}_\lambda^{\mathbf{s},\mathbf{t}}(x,b) + \lambda s_x.
\end{aligned} \tag{S26}
$$

Thus, by replacing $\sum_a P(a,y)$ and $\sum_b P(a,y)$ in Eq.(S25), $P(x,y)$ can be expressed as

$$\begin{aligned} P(x,y) =&\hat{P}_\lambda^{\mathbf{s},\mathbf{t}}(x,y) + \frac{\lambda}{1-\lambda}s_x\left(\sum_a \hat{P}_\lambda^{\mathbf{s},\mathbf{t}}(a,y)\right) + \frac{\lambda}{1-\lambda}t_y\left(\sum_b \hat{P}_\lambda^{\mathbf{s},\mathbf{t}}(x,b)\right) + \lambda^2 s_x t_y \\ =&\left(\hat{P}_\lambda^{\mathbf{s},\mathbf{t}}(x,y) - \frac{1}{(1-\lambda)^2}\sum_a \hat{P}_\lambda^{\mathbf{s},\mathbf{t}}(a,y)\sum_b \hat{P}_\lambda^{\mathbf{s},\mathbf{t}}(x,b)\right) \\ &+ \left(\frac{1}{1-\lambda}\sum_a \hat{P}_\lambda^{\mathbf{s},\mathbf{t}}(a,y) + \lambda t_y\right)\left(\frac{1}{1-\lambda}\sum_b \hat{P}_\lambda^{\mathbf{s},\mathbf{t}}(x,b) + \lambda s_x\right) \\ =&\left(\hat{P}_\lambda^{\mathbf{s},\mathbf{t}}(x,y) - \frac{1}{(1-\lambda)^2}\sum_a \hat{P}_\lambda^{\mathbf{s},\mathbf{t}}(a,y)\sum_b \hat{P}_\lambda^{\mathbf{s},\mathbf{t}}(x,b)\right) + \sum_a P(a,y)\sum_b P(x,b). \end{aligned} \tag{S27}$$

Then for all x, y, it holds that

$$\begin{aligned} \frac{1}{(1-\lambda)^2}\sum_a \hat{P}_\lambda^{\mathbf{s},\mathbf{t}}(a,y)\sum_b \hat{P}_\lambda^{\mathbf{s},\mathbf{t}}(x,b) &= \hat{P}_\lambda^{\mathbf{s},\mathbf{t}}(a,b) - \left(P(x,y) - \sum_a P(a,y)\sum_b P(x,b)\right) \\ &\geq -\left(P(x,y) - \sum_a P(a,y)\sum_b P(x,b)\right). \end{aligned} \tag{S28}$$

Note that $\sum_a \hat{P}_\lambda^{\mathbf{s},\mathbf{t}}(a,y)\sum_b \hat{P}_\lambda^{\mathbf{s},\mathbf{t}}(x,b) \geq 0$ and $\sum_{a,b} \hat{P}_\lambda^{\mathbf{s},\mathbf{t}}(a,b) = (1-\lambda)^2$. Then for any permutation $\varphi \in S_n$, we have

$$\begin{aligned} 2 &\geq \frac{1}{(1-\lambda)^2}\sum_x\left(\sum_a \hat{P}_\lambda^{\mathbf{s},\mathbf{t}}(a,\varphi(x)) + \sum_b \hat{P}_\lambda^{\mathbf{s},\mathbf{t}}(x,b)\right) \\ &\geq \frac{2}{(1-\lambda)^2}\sum_x\sqrt{\sum_a \hat{P}_\lambda^{\mathbf{s},\mathbf{t}}(a,\varphi(x))\sum_b \hat{P}_\lambda^{\mathbf{s},\mathbf{t}}(x,b)} \\ &\geq 2\sum_x\sqrt{\max\left\{0, -\frac{1}{(1-\lambda)^2}\left(P(x,\varphi(x)) - \sum_a P(a,\varphi(x))\sum_b P(x,b)\right)\right\}} \\ &= \frac{2}{1-\lambda}\sum_x\sqrt{\max\left\{0, \sum_a P(a,\varphi(x))\sum_b P(x,b) - P(x,\varphi(x))\right\}}. \end{aligned} \tag{S29}$$

This means that

$$\lambda \leq 1 - \sum_{x=1}^n\sqrt{\max\left\{0, \sum_b P(x,b)\sum_a P(a,\varphi(x)) - P(x,\varphi(x))\right\}}. \tag{S30}$$

Section S4. Proof for Eq.(13)

We restate the conclusion in Eq.(13): It holds that

$$\mathrm{C}_\lambda(P) \leq \mathrm{rank}_{\mathrm{psd}}^{\mathcal{E}_\lambda}(P). \tag{S31}$$

Proof: To show why this is the case, suppose $r \times r$ PSD matrices $\{C_i\}, \{D_j\}$ are an $\mathcal{E}_\lambda$-PSD factorization of P. Note that $\{C_i\}, \{D_j\}$ are also a PSD factorization of P. We assume that $\{\widetilde{C_i}\}, \{\widetilde{D_j}\}$ is a diagonal PSD factorization equivalent to $\{C_i\}, \{D_j\}$. Recall that by equivalence we mean that there exists an invertible H such that $\widetilde{C_i} = HC_iH^\dagger$ and $\widetilde{D_j} = (H^\dagger)^{-1} D_j H^{-1}$. It turns out that such an H always exists (*53*). We can verify that $\{\widetilde{C_i}\}, \{\widetilde{D_j}\}$ are also an $\mathcal{E}_\lambda$-PSD factorization for P, since $\{\widetilde{C_i}\}, \{\widetilde{D_j}\}$ are also a PSD factorization of P, and

$$\begin{aligned}
&\widetilde{C_i} - \frac{\lambda}{r}\mathrm{Tr}\left(\widetilde{C_i}\left(\sum_{k=1}^n \widetilde{C_k}\right)^{-1}\right)\sum_{k=1}^n \widetilde{C_k} \\
=&H\left(C_i - \frac{\lambda}{r}\mathrm{Tr}\left(HC_iH^\dagger\left(\sum_{k=1}^n HC_kH^\dagger\right)^{-1}\right)\sum_{k=1}^n C_k\right)H^\dagger \\
=&H\left(C_i - \frac{\lambda}{r}\mathrm{Tr}\left(C_i\left(\sum_{k=1}^n C_k\right)^{-1}\right)\sum_{k=1}^n C_k\right)H^\dagger
\end{aligned} \tag{S32}$$

is a PSD matrix (a similar conclusion holds for $\widetilde{D_j}$). Based on the above discussion, without loss of generality, we can suppose $\sum_k C_k = \sum_k D_k = \Lambda$, where $\Lambda = \mathrm{diag}(\beta_1, \ldots, \beta_r)$ is an invertible diagonal matrix and $\mathrm{Tr}\left(\Lambda^2\right) = 1$.

Let

$$\begin{aligned}
M_i &= \frac{1}{1-\lambda}\sqrt{\Lambda^{-1}}\left(C_i^T - \frac{\lambda}{r}\mathrm{Tr}\left(C_i\Lambda^{-1}\right)\Lambda\right)\sqrt{\Lambda^{-1}}, \\
N_j &= \frac{1}{1-\lambda}\sqrt{\Lambda^{-1}}\left(D_j - \frac{\lambda}{r}\mathrm{Tr}\left(D_j\Lambda^{-1}\right)\Lambda\right)\sqrt{\Lambda^{-1}}, \\
|\psi_\Lambda\rangle &= \sqrt{\Lambda} \otimes \sqrt{\Lambda}\sum_{k=1}^r |kk\rangle = \sum_{k=1}^r \beta_k |kk\rangle.
\end{aligned} \tag{S33}$$

We have that $\{M_i\}, \{N_j\}$ are valid POVMs, and $|\psi_\Lambda\rangle$ is a state. Furthermore, it holds that $\mathrm{Tr}\left(M_i\right) = \mathrm{Tr}\left(C_i\Lambda^{-1}\right)$ and $\mathrm{Tr}\left(N_j\right) = \mathrm{Tr}\left(D_j\Lambda^{-1}\right)$. In addition, it can be verified that $\mathcal{E}_\lambda(M_i) = \sqrt{\Lambda^{-1}}C_i^T\sqrt{\Lambda^{-1}}$

and $\mathcal{E}_\lambda(N_j) = \sqrt{\Lambda^{-1}} D_j \sqrt{\Lambda^{-1}}$. Then we have that

$$\begin{aligned}
&\mathrm{Tr}\left(\mathcal{E}_\lambda \otimes \mathcal{E}_\lambda(|\psi_\Lambda\rangle\langle\psi_\Lambda|) M_i \otimes N_j\right)\\
=&\mathrm{Tr}\left(|\psi_\Lambda\rangle\langle\psi_\Lambda| \mathcal{E}_\lambda(M_i) \otimes \mathcal{E}_\lambda(N_j)\right)\\
=&\mathrm{Tr}\left(|\psi_\Lambda\rangle\langle\psi_\Lambda| \sqrt{\Lambda^{-1}} C_i^T \sqrt{\Lambda^{-1}} \otimes \sqrt{\Lambda^{-1}} D_j \sqrt{\Lambda^{-1}}\right)\\
=&\mathrm{Tr}\left(\sum_{k=1}^{r} |kk\rangle \sum_{k=1}^{r} \langle kk| C_i^T \otimes D_j\right)\\
=&\mathrm{Tr}\left(C_i D_j\right)\\
=&P_{ij}
\end{aligned} \tag{S34}$$

That is, $|\psi_\Lambda\rangle$ can generate P under noise $\mathcal{E}_\lambda \otimes \mathcal{E}_\lambda$, which means the minimum local dimension of a seed state for P under noise $\mathcal{E}_\lambda \otimes \mathcal{E}_\lambda$ is no more than $\mathrm{rank}_{\mathrm{psd}}^{\mathcal{E}_\lambda}(P)$.

Section S5. Proofs for Eq.(14) and Eq.(15)

We restate Eq.(14) and Eq.(15): Given $P \in \mathbb{R}_{>0}^{n\times n}$ and $\lambda \geq 0$, if there exist $\mathbf{s}, \mathbf{t} \in \mathbb{R}_{>0}^n$ with $\|\mathbf{s}\|_1 = \|\mathbf{t}\|_1 = 1$ such that $\hat{P}_\lambda^{\mathbf{s},\mathbf{t}}(x, y) \geq 0$ for all x, y, then

$$\mathrm{C}_\lambda(P) \leq \mathrm{rank}_{\mathrm{psd}}(\hat{P}_\lambda^{\mathbf{s},\mathbf{t}}) \left\lceil \frac{1}{\min_{x,y}\{s_x, t_y\}} \right\rceil, \tag{S35}$$

and

$$\mathrm{C}_\lambda(P) \geq \frac{1}{1-\lambda} \left(\inf_{\mathbf{s}',\mathbf{t}'} \max_{x,y} \left\{ \frac{\sum_b P(x,b)}{s'_x}, \frac{\sum_a P(a,y)}{t'_y} \right\} - \lambda \right), \tag{S36}$$

where $\mathbf{s}', \mathbf{t}' \in \mathbb{R}_{>0}^n$ with $\|\mathbf{s}'\|_1 = \|\mathbf{t}'\|_1 = 1$ satisfy $\hat{P}_\lambda^{\mathbf{s}',\mathbf{t}'}(x, y) \geq 0$.

Proof: (Proof of Eq.(14))

As shown in the proof for Proposition 1, if there exist $\mathbf{s}, \mathbf{t} \in \mathbb{R}_{>0}^n$ with $\|\mathbf{s}\|_1 = \|\mathbf{t}\|_1 = 1$ such that $\hat{P}_\lambda^{\mathbf{s},\mathbf{t}}(x, y) \geq 0$ for all x, y, one can find POVMs $\{E'_x\}$ and $\{F'_y\}$, and a quantum state $\sigma' \in D(\mathbb{C}^{d'} \otimes \mathbb{C}^{d'})$ such that $\mathrm{Tr}\left(E'_x \otimes F'_y \sigma'\right) = \frac{1}{(1-\lambda)^2} \hat{P}_\lambda^{\mathbf{s},\mathbf{t}}(x, y)$, where $d' = \mathrm{rank}_{\mathrm{psd}}(\hat{P}_\lambda^{\mathbf{s},\mathbf{t}})$.

By taking $k \in \mathbb{Z}^+$ such that

$$\begin{aligned}
d' k s_x - \mathrm{Tr}\left(E'_x\right) \geq 0,\\
d' k t_y - \mathrm{Tr}\left(F'_y\right) \geq 0,
\end{aligned} \tag{S37}$$

for all x, y, we can construct a $d \times d$ seed state $\sigma = |0\rangle\langle 0| \otimes \sigma' \otimes |0\rangle\langle 0|$, $|0\rangle\langle 0| \in D(\mathbb{C}^k)$, and

POVMs

$$
\begin{aligned}
E_x &= |0\rangle\langle 0| \otimes E'_x + \left(\frac{d'ks_x - \mathrm{Tr}\left(E'_x\right)}{k-1}\right)(I_k - |0\rangle\langle 0|) \otimes \frac{I_{d'}}{d'},\\
F_y &= F'_y \otimes |0\rangle\langle 0| + \left(\frac{d'kt_y - \mathrm{Tr}\left(F'_y\right)}{k-1}\right)\frac{I_{d'}}{d'} \otimes (I_k - |0\rangle\langle 0|),
\end{aligned} \tag{S38}
$$

to generate P, i.e., $P(x,y) = \mathrm{Tr}\left(E_x \otimes F_y \mathcal{E}_\lambda \otimes \mathcal{E}_\lambda(\sigma)\right)$, where $d = d'k$.

In the above process, the dimension for the seed state is $d = d'k = \mathrm{rank}_{\mathrm{psd}}(\hat{P}^{\mathbf{s},\mathbf{t}}_\lambda)k$, thus we have $\mathrm{C}_\lambda(P) \leq \mathrm{rank}_{\mathrm{psd}}(\hat{P}^{\mathbf{s},\mathbf{t}}_\lambda)k$. Actually, to ensure that Eq.(S37) holds, it suffices to take

$$
k = \left\lceil \max_{x,y}\left\{\frac{\mathrm{Tr}\left(E'_x\right)}{\mathrm{rank}_{\mathrm{psd}}(\hat{P}^{\mathbf{s},\mathbf{t}}_\lambda)}\frac{1}{s_x}, \frac{\mathrm{Tr}\left(F'_y\right)}{\mathrm{rank}_{\mathrm{psd}}(\hat{P}^{\mathbf{s},\mathbf{t}}_\lambda)}\frac{1}{t_y}\right\}\right\rceil \leq \left\lceil \frac{1}{\min_{x,y}\{s_x, t_y\}}\right\rceil. \tag{S39}
$$

(Proof of Eq.(15))

Suppose for a local dimension d, a quantum state $\sigma \in D(\mathbb{C}^d \otimes \mathbb{C}^d)$, and POVMs $\{E_x, F_y\}$, it holds that $P(x,y) = \mathrm{Tr}\left(E_x \otimes F_y \mathcal{E}_\lambda \otimes \mathcal{E}_\lambda(\sigma)\right)$. Let $s_x = \mathrm{Tr}\left(E_x\right)/d$ and $t_y = \mathrm{Tr}\left(F_y\right)/d$. By Proposition 1 we have that $\hat{P}^{\mathbf{s},\mathbf{t}}_\lambda(x,y)$ defined in Eq.(4) is nonnegative for all x, y. Denote $\sigma_A = \mathrm{Tr}_B(\sigma)$ and $\sigma_B = \mathrm{Tr}_A(\sigma)$. For arbitrary x, we have

$$
\begin{aligned}
\sum_b P(x,b) &= \mathrm{Tr}\left(E_x \mathcal{E}_\lambda(\sigma_A)\right) = (1-\lambda)\mathrm{Tr}\left(E_x\sigma_A\right) + \lambda\frac{\mathrm{Tr}\left(E_x\right)}{d}\\
&\leq (1-\lambda)\mathrm{Tr}\left(E_x\right) + \lambda\frac{\mathrm{Tr}\left(E_x\right)}{d} = (d(1-\lambda)+\lambda)s_x,
\end{aligned} \tag{S40}
$$

thus $s_x \geq \frac{\sum_b P(x,b)}{d(1-\lambda)+\lambda}$, which leads to $d \geq \frac{1}{1-\lambda}\left(\frac{\sum_b P(x,b)}{s_x} - \lambda\right)$. Similarly, for arbitrary y, we can obtain $d \geq \frac{1}{1-\lambda}\left(\frac{\sum_a P(a,y)}{t_y} - \lambda\right)$, which leads to

$$
d \geq \frac{1}{1-\lambda}\left(\max_{x,y}\left\{\frac{\sum_b P(x,b)}{s_x}, \frac{\sum_a P(a,y)}{t_y}\right\} - \lambda\right). \tag{S41}
$$

On both sides of Eq.(S41), taking the infimum over all possible quantum protocols that can generate P, we get

$$
\mathrm{C}_\lambda(P) \geq \frac{1}{1-\lambda}\left(\inf_{\mathbf{s}',\mathbf{t}'}\max_{x,y}\left\{\frac{\sum_b P(x,b)}{s'_x}, \frac{\sum_a P(a,y)}{t'_y}\right\} - \lambda\right). \tag{S42}
$$

Section S6. Some properties of A_m and B_m

Recall that $A_m \in \mathbb{R}^{(m+1)\times(m+1)}$ is a classical correlation with the entries given by

$$A_m = \begin{pmatrix} \frac{(1-q)^2}{2} & \frac{q(1-q)}{2m} & \cdots & \frac{q(1-q)}{2m} \\ \frac{q(1-q)}{2m} & & & \\ \vdots & & \frac{1+q^2}{2} B_m & \\ \frac{q(1-q)}{2m} & & & \end{pmatrix}, \tag{S43}$$

where $k \in (0,1)$ is a variable, $q = \frac{1}{1-k} - \sqrt{\frac{1}{(1-k)^2} - 1}$, and $B_m \in \mathbb{R}^{m\times m}$ is also a classical correlation given by

$$B_m(x,y) = \frac{1}{m^2}\left(1 - k\cos\left(2\pi\frac{x-1+y-1}{m}\right)\right), x, y = 1, 2, \cdots, m. \tag{S44}$$

As shown in (Fig. 1) of the main text, B_m represents the slack matrix associated with two concentric regular polygons, denoted by R_{out} and R_{in} respectively. Here R_{out} has an inscribed circle with a radius of 1, while R_{in} has a circumscribed circle with a radius $k < 1$. The vertices of R_{in} are positioned at the midpoints of the sides of R_{out}. More details about the slack matrix can be found in (*47*).

The outer polygon can be expressed as $R_{\text{out}} = \{v \in \mathbb{R}^2 | a_x^T v \leq 1, x = 1, \cdots, m\}$, where

$$a_x = \left(\cos\frac{2(x-1)\pi}{m}, \sin\frac{2(x-1)\pi}{m}\right) \tag{S45}$$

is the midpoint of the x-th edge of the large polygon. The y-th vertex of the smaller polygon R_{in} is

$$b_y = \left(k\cos\frac{2(y-1)\pi}{m}, -k\sin\frac{2(y-1)\pi}{m}\right). \tag{S46}$$

Thus the slack matrix between R_{out} and R_{in} is $S_{B,A} = (1 - a_x b_y^T)_{xy} = \left[1 - k\cos\left(2\pi\frac{x-1+y-1}{m}\right)\right]_{x,y=1,\cdots,m}$, which leads to $B_m = \frac{1}{m^2} S_{B,A}$.

Lemma S1 *For B_m defined above, we have*

1. $\text{rank}_+(B_m) > \log_2(m/2)$, *when* $k > \frac{\cos(2\pi/m)}{\cos^2(\pi/m)}$,
2. $\text{rank}_{\text{psd}}(B_m) = 2$.

Proof: (Part 1)

By the geometric interpretation of B_m we can derive a lower bound for $\mathrm{rank}_+(B_m)$. We first show that $\mathrm{rank}_+(B_m) > \log_2 l$ if $k > \frac{\cos(\pi/l)}{\cos^2(\pi/m)}$.

A lower bound for $\mathrm{rank}_+(B_m)$ is derived from $\mathrm{rank}^*_+(B_m)$, known as the restricted nonnegative rank. This quantity represents the minimum number k of vertices of a convex polygon $T \in \mathbb{R}^2$ such that $R_{\mathrm{in}} \subset T \subset R_{\mathrm{out}}$, where R_{out} and R_{in} denote the polygons described in Eq.(S45) and (S46) respectively. More details can be found in (*52*).

A necessary condition for an l-gon T to be put between R_{in} and R_{out} is $R^C_{\mathrm{in}} \subset T \subset R^C_{\mathrm{out}}$, where R^C_{out} denotes the circumscribed circle of R_{out} with a radius of $\frac{1}{\cos\frac{\pi}{m}}$, and R^C_{in} denotes the inscribed circle of R_{in} with a radius of $k\cos\frac{\pi}{m}$. Therefore, every angle of such a T is at least $2\arcsin(k\cos^2\frac{\pi}{m})$, while the smallest angle of any l-gon is at most $\pi - \frac{2\pi}{l}$. Consequently, it must hold that $k \le \frac{\cos(\pi/l)}{\cos^2(\pi/m)}$. In other words, if $k > \frac{\cos(\pi/l)}{\cos^2(\pi/m)}$, no l-gon can be put between R_{in} and R_{out}. Thus, $\mathrm{rank}^*_+(B_m) > l$. According to Theorem 6 in (*52*), we have that $\mathrm{rank}_+(B_m) \ge \log_2(\mathrm{rank}^*_+(B_m)) > \log_2(l)$.

By taking $l = m/2$, it follows that $\mathrm{rank}_+(B_m) > \log_2(l) = \log_2(m/2)$ when $k > \frac{\cos(\pi/l)}{\cos^2(\pi/m)} = \frac{\cos(2\pi/m)}{\cos^2(\pi/m)}$, where $\frac{\cos(2\pi/m)}{\cos^2(\pi/m)} < 1$. Thus, for any $m \ge 3$, we can always choose k close enough to 1 such that $\mathrm{rank}_+(B_m) > \log_2(m/2)$.

(Part 2)

We can take

$$\begin{aligned} C_x &= \frac{1}{\sqrt{2m}}\begin{pmatrix} 1 & \sqrt{k}e^{\frac{x-1}{m}2\pi i} \\ \sqrt{k}e^{-\frac{x-1}{m}2\pi i} & 1 \end{pmatrix}, \\ D_y &= \frac{1}{\sqrt{2m}}\begin{pmatrix} 1 & -\sqrt{k}e^{-\frac{y-1}{m}2\pi i} \\ -\sqrt{k}e^{\frac{y-1}{m}2\pi i} & 1 \end{pmatrix}, \end{aligned} \tag{S47}$$

where $i = \sqrt{-1}$. It is straightforward to check $B_m(x,y) = \mathrm{Tr}\left(C_x D_y\right)$ for all $x, y = 1, \cdots, m$, which means that $\mathrm{rank}_{\mathrm{psd}}(B_m) \le 2$. Also note that $\mathrm{rank}(B_m) \ge 2$, thus we have that $\mathrm{rank}_{\mathrm{psd}}(B_m) = 2$. Now we complete the proof.

Based on the properties of B_m, we can now characterize A_m accordingly.

Lemma S2 *For A_m defined in Eq.*(16)*, we have*

1. $\mathrm{rank}_+(A_m) > \log_2(m/2)$ *when* $k > \frac{\cos(2\pi/m)}{\cos^2(\pi/m)}$,

2. $\mathrm{rank}_{\mathrm{psd}}(A_m) \le 3$.

Proof: (Part 1)

Since B_m is a submatrix of A_m, we have $\mathrm{rank}_+(A_m) \geq \mathrm{rank}_+(B_m)$. Meanwhile, according to Lemma S1, it holds that $\mathrm{rank}_+(B_m) > \log_2(m/2)$ if $k > \frac{\cos(2\pi/m)}{\cos^2(\pi/m)}$.

(Part 2)

We take the same $\{C_x, D_y\}$ as in Eq.(S47), then let

$$\begin{aligned} C'_1 = D'_1 &= \frac{1-q}{2\sqrt{1+q^2}} \begin{pmatrix} \sqrt{2} & & \\ & q & \\ & & q \end{pmatrix}, \\ C'_x &= \sqrt{\frac{1+q^2}{2}} \begin{pmatrix} 0 & \\ & C_{x-1} \end{pmatrix} \text{ for } 2 \leq x \leq m+1, \\ D'_y &= \sqrt{\frac{1+q^2}{2}} \begin{pmatrix} 0 & \\ & D_{y-1} \end{pmatrix} \text{ for } 2 \leq y \leq m+1. \end{aligned} \tag{S48}$$

We have

$$\begin{aligned} \mathrm{Tr}\left(C'_1 D'_1\right) &= \frac{(1-q)^2}{2}, \\ \mathrm{Tr}\left(C'_x D'_1\right) = \mathrm{Tr}\left(C'_1 D'_y\right) &= \frac{q(1-q)}{2m} \text{ for } 2 \leq x, y \leq m+1, \\ \mathrm{Tr}\left(C'_x D'_y\right) &= \frac{1+q^2}{2}\mathrm{Tr}\left(C_{x-1} D_{y-1}\right) = \frac{1+q^2}{2}(P_{m,k})_{x-1,y-1} \text{ for } 2 \leq x, y \leq m+1. \end{aligned} \tag{S49}$$

Thus C'_x, D'_y is a PSD decomposition of A_m, implying that $\mathrm{rank}_{\mathrm{psd}}(A_m) \leq 3$.

Section S7. Proof for Theorem 2

Theorem S2 *As the noise strength λ approaches q, the quantum advantage in generating A_m decreases asymptotically to* 0.

Proof: Let $\lambda = q - \epsilon$, where $0 < \epsilon < q$. And still use the notation

$$\hat{P}_\lambda^{\mathbf{s},\mathbf{t}}(x, y) = A_m(x, y) - \lambda s_x \sum_{a=1}^{m+1} A_m(a, y) - \lambda t_y \sum_{b=1}^{m+1} A_m(x, b) + \lambda^2 s_x t_y. \tag{S50}$$

According to Proposition 1, there exists ρ such that $\rho \xrightarrow{q-\epsilon} P$ if and only if there exist $\mathbf{s}, \mathbf{t} \in \mathbb{R}_{>0}^{m+1}$ with $\|\mathbf{s}\|_1 = \|\mathbf{t}\|_1 = 1$ such that $\hat{P}_\lambda^{\mathbf{s},\mathbf{t}}(x, y) \geq 0$.

First, we prove that for any $0 < \lambda < q$, A_m can always be generated by some quantum state. Let

$$\mathbf{s} = \mathbf{t} = \big(\eta, (1-\eta)/m, (1-\eta)/m, \cdots, (1-\eta)/m\big), \tag{S51}$$

where $\eta = \min\left\{\frac{1-q}{2(q-\epsilon)}, \frac{\epsilon(1+q)}{2q(q-\epsilon)}\right\}$. Note that $B_m(x, y) \geq \frac{1-k}{m^2}$. For $2 \leq x, y \leq m+1$, we have that

$$\begin{aligned}
\hat{P}_\lambda^{\mathbf{s},\mathbf{t}}(1,1) \geq& \frac{(1-q)^2}{2} - (q-\epsilon)\eta(1-q) \geq 0,\\
\hat{P}_\lambda^{\mathbf{s},\mathbf{t}}(1,y) =& \hat{P}_\lambda^{\mathbf{s},\mathbf{t}}(x,1) \geq \frac{q(1-q)}{2m} - (q-\epsilon)\eta\frac{1+q}{2m} - (q-\epsilon)\frac{1-\eta}{m}\frac{1-q}{2} \geq 0,\\
\hat{P}_\lambda^{\mathbf{s},\mathbf{t}}(x,y) \geq& \frac{1+q^2}{2}\frac{1-k}{m^2} - 2(q-\epsilon)\frac{1-\eta}{m}\frac{1+q}{2m} + (q-\epsilon)^2\frac{(1-\eta)^2}{m^2}\\
\geq& \frac{1+q^2}{2}\frac{1-k}{m^2} - 2q\frac{1}{m}\frac{1+q}{2m} + q^2\frac{1}{m^2}\\
=& \frac{1}{m^2}\left(\frac{(1+q^2)(1-k)}{2} - q\right) = 0.
\end{aligned} \tag{S52}$$

That is, when $0 < \lambda < q$, $\hat{P}_\lambda^{\mathbf{s},\mathbf{t}}(x, y) \geq 0$ for any x, y, which means there exists a quantum state ρ such that $\rho \xrightarrow{\lambda} A_m$.

By Eq.(14), if ϵ is small enough, we can obtain

$$\mathrm{C}_\lambda(A_m) \leq \mathrm{rank}_{\mathrm{psd}}(\hat{P}_\lambda^{\mathbf{s},\mathbf{t}}) \left\lceil \frac{1}{\min_{x,y}\{s_x, t_y\}} \right\rceil \leq m \left\lceil \frac{2q(q-\epsilon)}{\epsilon(1+q)} \right\rceil = O(\epsilon^{-1}). \tag{S53}$$

Second, we now show that when $\epsilon \to 0$, for any $\mathbf{s}, \mathbf{t} \in \mathbb{R}_{>0}^{m+1}$ satisfying $\|\mathbf{s}\|_1 = \|\mathbf{t}\|_1 = 1$ and

$\hat{P}_\lambda^{\mathbf{s},\mathbf{t}}(x, y) \geq 0$ for all x, y, we must have $\min\{s_1, t_1\} \to 0$. In fact, it holds that

$$\begin{aligned}
&\sum_{i=2}^{m+1} \hat{P}_\lambda^{\mathbf{s},\mathbf{t}}(i,1) + \sum_{j=2}^{m+1} \hat{P}_\lambda^{\mathbf{s},\mathbf{t}}(1,j) \\
=&\sum_{i=2}^{m+1} A_m(i,1) - \sum_{i=2}^{m+1} \lambda s_i \sum_{a=1}^{m+1} A_m(a,1) - \sum_{i=2}^{m+1} \lambda t_1 \sum_{b=1}^{m+1} A_m(i,b) + \sum_{i=2}^{m+1} \lambda^2 s_i t_1 \\
&+ \sum_{j=2}^{m+1} A_m(1,j) - \sum_{j=2}^{m+1} \lambda s_1 \sum_{a=1}^{m+1} A_m(a,j) - \sum_{j=2}^{m+1} \lambda t_j \sum_{b=1}^{m+1} A_m(1,b) + \sum_{j=2}^{m+1} \lambda^2 s_1 t_j \\
=&\frac{q(1-q)}{2} - \lambda(1-s_1)\frac{1-q}{2} - \lambda t_1 \frac{1+q}{2} + \lambda^2(1-s_1)t_1 \\
&+ \frac{q(1-q)}{2} - \lambda s_1 \frac{1+q}{2} - \lambda(1-t_1)\frac{1-q}{2} + \lambda^2 s_1(1-t_1) \\
\leq&\frac{q(1-q)}{2} - \lambda(1-s_1)\frac{1-q}{2} - \lambda t_1 \frac{1+q}{2} + q^2(1-s_1)t_1 \\
&+ \frac{q(1-q)}{2} - \lambda s_1 \frac{1+q}{2} - \lambda(1-t_1)\frac{1-q}{2} + q^2 s_1(1-t_1) \\
=&-2q^2 s_1 t_1 + \epsilon - \epsilon(1-s_1-t_1)q \\
\leq&-2q^2 s_1 t_1 + \epsilon(1+q).
\end{aligned} \tag{S54}$$

Therefore, for any $\mathbf{s}, \mathbf{t} \in \mathbb{R}_{>0}^{m+1}$ and $\|\mathbf{s}\|_1 = \|\mathbf{t}\|_1 = 1$, to let $\hat{P}_\lambda^{\mathbf{s},\mathbf{t}}(x, y) \geq 0$, we must have

$$\min\{s_1, t_1\} \leq \sqrt{\frac{\epsilon(1+q)}{2q^2}}. \tag{S55}$$

Then according to Eq.(15),

$$\begin{aligned}
\mathrm{C}_\lambda(A_m) &\geq \frac{1}{1-\lambda}\left(\inf_{\mathbf{s},\mathbf{t}} \max_{x,y} \left\{\frac{\sum_b A_m(x,b)}{s_x}, \frac{\sum_a A_m(a,y)}{t_y}\right\} - \lambda\right) \\
&\geq \frac{1}{1-\lambda}\left(\inf_{\mathbf{s},\mathbf{t}} \max \left\{\frac{\sum_b A_m(1,b)}{s_1}, \frac{\sum_a A_m(a,1)}{t_1}\right\} - \lambda\right) \\
&\geq \frac{1}{1-q+\epsilon}\left(\frac{1-q}{2}\sqrt{\frac{2q^2}{\epsilon(1+q)}} - q + \epsilon\right) = \Omega(\epsilon^{-1/2}).
\end{aligned} \tag{S56}$$

Also note that $\mathrm{R}(A_m)$ is independent of ϵ. In conclusion, when ϵ is small, we have that

$$\mathcal{S}_\lambda(A_m) = \frac{\mathrm{R}(A_m)}{\lceil \log_2 \mathrm{C}_\lambda(A_m) \rceil} = \Theta\left(\frac{1}{\log \epsilon^{-1}}\right). \tag{S57}$$

In other words, when $\lambda \to q$, $\mathrm{C}_\lambda(A_m) \to \infty$, implying that $\mathcal{S}_\lambda(A_m) \to 0$.

Section S8. Mathematical characterization for sudden death

We prove the following result, which is equivalent to Theorem 4.

Theorem S3 *For any correlation $P \in \mathbb{R}_{>0}^{n\times n}$, the following statements are equivalent:*

1. $\sup_{\lambda\in\Lambda(P)} \mathrm{C}_\lambda(P) = \infty$.
2. *$\Lambda(P)$ is a right open interval.*
3. *There exists $\lambda > 0$, such that there exist $\mathbf{s}, \mathbf{t} \in \mathbb{R}_{\geq 0}^n$ with $\|\mathbf{s}\|_1 = \|\mathbf{t}\|_1 = 1$ such that $\hat{P}_\lambda^{\mathbf{s},\mathbf{t}}(x, y)$ defined in Eq.(4) is nonnegative for all x, y. However, P is not reachable for any quantum protocols under noise $\mathcal{E}_\lambda \otimes \mathcal{E}_\lambda$.*

Proof: "1 $\Rightarrow$ 2": If $\Lambda(P)$ is right closed, we choose $\lambda_u = \sup \Lambda(P) \in \Lambda(P)$. Thus, there exist $\mathbf{s}, \mathbf{t} \in \mathbb{R}_{>0}^n$ with $\|\mathbf{s}\|_1 = \|\mathbf{t}\|_1 = 1$ such that $\hat{P}_{\lambda_u}^{\mathbf{s},\mathbf{t}}(x, y) \geq 0$. According to Eq.(14), $\mathrm{C}_{\lambda_u}(P)$ has an upper bound.

Since for any $\lambda \leq \lambda_u$, it holds that $\mathrm{C}_\lambda(P) \leq \mathrm{C}_{\lambda_u}(P)$, i.e., Alice and Bob can simulate strong noise with weak noise by introducing more noise. Then we have that $\sup_{\lambda\in\Lambda(P)} \mathrm{C}_\lambda(P)$ has an upper bound, which contradicts $\sup_{\lambda\in\Lambda(P)} \mathrm{C}_\lambda(P) = \infty$.

"2 $\Rightarrow$ 3": Let

$$ST_\lambda = \left\{(\mathbf{s}, \mathbf{t}) \in \mathbb{R}_{\geq 0}^n \times \mathbb{R}_{\geq 0}^n \middle| \hat{P}_\lambda^{\mathbf{s},\mathbf{t}} \geq 0, \|\mathbf{s}\|_1 = \|\mathbf{t}\|_1 = 1\right\}. \tag{S58}$$

Note that in this definition we allow the entries of $\mathbf{s}$ and $\mathbf{t}$ to be 0, thus ST_λ is closed and $ST_\lambda \subset \left\{(\mathbf{s}, \mathbf{t}) \in \mathbb{R}_{\geq 0}^n \times \mathbb{R}_{\geq 0}^n \middle| \|\mathbf{s}\|_1 = \|\mathbf{t}\|_1 = 1\right\}$ which is a compact set. Also $\forall\ 0 \leq \lambda \leq \lambda' \in \Lambda(P)$, $ST_\lambda \supseteq ST_{\lambda'} \neq \emptyset$, which follows directly from Eq.(7). So, we have $\bigcap_{\lambda\in\Lambda(P)} ST_\lambda \neq \emptyset$.

For any $\lambda \in \Lambda(P)$ and $(\mathbf{s}', \mathbf{t}') \in \bigcap_{\lambda\in\Lambda(P)} ST_\lambda$, we always have $\hat{P}_\lambda^{\mathbf{s}',\mathbf{t}'}(x, y) \geq 0$ for all x, y. Then $\hat{P}_{\sup\Lambda(P)}^{\mathbf{s}',\mathbf{t}'}(x, y) = \lim_{\lambda\to\sup\Lambda(P)} \hat{P}_\lambda^{\mathbf{s}',\mathbf{t}'}(x, y) \geq 0$. That is, for $\lambda_u = \sup \Lambda(P)$, there exist $\mathbf{s}, \mathbf{t} \in \mathbb{R}_{\geq 0}^n$ with $\|\mathbf{s}\|_1 = \|\mathbf{t}\|_1 = 1$ such that $\hat{P}_{\lambda_u}^{\mathbf{s},\mathbf{t}}(x, y) \geq 0$. However, since $\sup \Lambda(P) \notin \Lambda(P)$, there do not exist $\mathbf{s}, \mathbf{t} \in \mathbb{R}_{>0}^n$ with $\|\mathbf{s}\|_1 = \|\mathbf{t}\|_1 = 1$ satisfying $\hat{P}_{\lambda_u}^{\mathbf{s},\mathbf{t}}(x, y) \geq 0$.

"3 $\Rightarrow$ 2": Suppose that λ_u satisfies Statement 3 and $\hat{P}_{\lambda_u}^{\mathbf{s}_u,\mathbf{t}_u}(x, y) \geq 0$ for all x, y, where $\mathbf{s}_u, \mathbf{t}_u \in \mathbb{R}_{\geq 0}^n$ satisfy $\|\mathbf{s}_u\|_1 = \|\mathbf{t}_u\|_1 = 1$. We prove that for any $\epsilon > 0$, $\lambda_u - \epsilon \in \Lambda(P)$.

For any x, y, if $(\mathbf{s}_u)_x = (\mathbf{t}_u)_y = 0$, then $\hat{P}_{\lambda-\epsilon}^{\mathbf{s}_u,\mathbf{t}_u}(x, y) = P(x, y) > 0$. And if $(\mathbf{s}_u)_x = 0, (\mathbf{t}_u)_y > 0$, we have

$$\frac{\partial \hat{P}_\lambda^{\mathbf{s}_u,\mathbf{t}_u}(x, y)}{\partial \lambda}|_{\lambda=\lambda_u-\epsilon} = -(\mathbf{t}_u)_y \sum_b P(x, b) < 0. \tag{S59}$$

Thus $\hat{P}^{\mathbf{s}_u,\mathbf{t}_u}_{\lambda_u-\epsilon}(x,y) > \hat{P}^{\mathbf{s}_u,\mathbf{t}_u}_{\lambda_u}(x,y) \geq 0$. The case that $(\mathbf{s}_u)_x > 0$ and $(\mathbf{t}_u)_y = 0$ is similar.

Lastly, if $(\mathbf{s}_u)_x > 0, (\mathbf{t}_u)_y > 0$, then

$$\frac{\partial \hat{P}^{\mathbf{s}_u,\mathbf{t}_u}_{\lambda}(x,y)}{\partial \lambda}|_{\lambda=\lambda_u-\epsilon} = \frac{\partial \hat{P}^{\mathbf{s}_u,\mathbf{t}_u}_{\lambda}(x,y)}{\partial \lambda}|_{\lambda=\lambda_u} - 2\epsilon(\mathbf{s}_u)_x(\mathbf{t}_u)_y \leq -2\epsilon(\mathbf{s}_u)_x(\mathbf{t}_u)_y < 0. \tag{S60}$$

Thus $\hat{P}^{\mathbf{s}_u,\mathbf{t}_u}_{\lambda_u-\epsilon}(x,y) > \hat{P}^{\mathbf{s}_u,\mathbf{t}_u}_{\lambda_u}(x,y) \geq 0$.

In conclusion, we always have $\hat{P}^{\mathbf{s}_u,\mathbf{t}_u}_{\lambda_u-\epsilon}(x,y) > 0$. Let $w = \min_{x,y} \hat{P}^{\mathbf{s}_u,\mathbf{t}_u}_{\lambda_u-\epsilon}(x,y) > 0$. Note that

$$\left|\hat{P}^{\mathbf{s},\mathbf{t}}_{\lambda}(x,y) - \hat{P}^{\mathbf{s}',\mathbf{t}'}_{\lambda}(x,y)\right| \leq 3\left|s_x - s'_x\right| + 3\left|t_y - t'_y\right| \tag{S61}$$

for any $\lambda, \mathbf{s}, \mathbf{t}, \mathbf{s}', \mathbf{t}', x, y$. Then for any $\mathbf{s}, \mathbf{t} \in \mathbb{R}^n_{>0}$ that satisfy $\|\mathbf{s} - \mathbf{s}_u\|_2 \leq \frac{w}{6}$ and $\|\mathbf{t} - \mathbf{t}_u\|_2 \leq \frac{w}{6}$ (such $\mathbf{s}, \mathbf{t}$ exist), we have

$$\hat{P}^{\mathbf{s},\mathbf{t}}_{\lambda_u-\epsilon}(x,y) \geq \hat{P}^{\mathbf{s}_u,\mathbf{t}_u}_{\lambda_u-\epsilon}(x,y) - w \geq 0. \tag{S62}$$

Thus $\lambda_u - \epsilon \in \Lambda(P)$, which means $\Lambda(P) = [0, \lambda_u)$.

"$2 \Rightarrow 1$": Let $\lambda_u = \sup \Lambda(P)$. Suppose $\sup_{\lambda \in \Lambda(P)} \mathrm{C}_\lambda(P) < d$ for some constant d. Then from Eq.(7), for any $\lambda \in \Lambda(P)$, there exist $\mathbf{s}, \mathbf{t} \in \mathbb{R}^n_{>0}$ such that $\|\mathbf{s}\|_1 = \|\mathbf{t}\|_1 = 1$ and $\hat{P}^{\mathbf{s},\mathbf{t}}_{\lambda}(x,y) \geq 0$ for all x, y, where $s_x \geq \frac{\sum_b P(x,b)}{d(1-\lambda)+\lambda}$ and $t_y \geq \frac{\sum_a P(a,y)}{d(1-\lambda)+\lambda}$, as shown in Eq.(S41). Let

$$v = \min_{x,y}\left\{\frac{\sum_b P(x,b)}{d(1-\lambda_u)+\lambda_u}, \frac{\sum_a P(a,y)}{d(1-\lambda_u)+\lambda_u}\right\}. \tag{S63}$$

We have $\mathbf{s}, \mathbf{t} \in \mathbb{R}^n_{\geq v}$. Again let

$$ST_\lambda = \left\{(\mathbf{s}, \mathbf{t}) \in \mathbb{R}^n_{\geq v} \times \mathbb{R}^n_{\geq v} \middle| \hat{P}^{\mathbf{s},\mathbf{t}}_{\lambda} \geq 0, \|\mathbf{s}\|_1 = \|\mathbf{t}\|_1 = 1\right\}. \tag{S64}$$

Note that ST_λ is closed and $ST_\lambda \subset \left\{(\mathbf{s}, \mathbf{t}) \in \mathbb{R}^n_{\geq v} \times \mathbb{R}^n_{\geq v} \middle| \|\mathbf{s}\|_1 = \|\mathbf{t}\|_1 = 1\right\}$ which is compact. And for any $\lambda \leq \lambda' \in \Lambda(P)$, $ST_\lambda \supseteq ST_{\lambda'} \neq \emptyset$, which is directly from Eq.(7). So, we have $\bigcap_{\lambda \in \Lambda(P)} ST_\lambda \neq \emptyset$.

For any $\lambda \in \Lambda(P)$ and $(\mathbf{s}', \mathbf{t}') \in \bigcap_{\lambda \in \Lambda(P)} ST_\lambda$, we always have $\hat{P}^{\mathbf{s}',\mathbf{t}'}_{\lambda}(x,y) \geq 0$ for all x, y. Then

$$\hat{P}^{\mathbf{s}',\mathbf{t}'}_{\lambda_u}(x,y) = \hat{P}^{\mathbf{s}',\mathbf{t}'}_{\sup \Lambda(P)}(x,y) = \lim_{\lambda \to \sup \Lambda(P)} \hat{P}^{\mathbf{s}',\mathbf{t}'}_{\lambda}(x,y) \geq 0. \tag{S65}$$

Thus $\sup \Lambda(P) \in \Lambda(P)$, which contradicts the fact that $\Lambda(P)$ is right open. Thus, we have $\sup_{\lambda \in \Lambda(P)} \mathrm{C}_\lambda(P) = \infty$.

REFERENCES AND NOTES

1. F. Arute, K. Arya, R. Babbush, D. Bacon, J. C. Bardin, R. Barends, R. Biswas, S. Boixo, F. G. Brandao, D. A. Buell, B. Burkett, Y. Chen, Z. Chen, B. Chiaro, R. Collins, W. Courtney, A. Dunsworth, E. Farhi, B. Foxen, A. Fowler, C. Gidney, M. Giustina, R. Graff, K. Guerin, S. Habegger, M. P. Harrigan, M. J. Hartmann, A. Ho, M. Hoffmann, T. Huang, T. S. Humble, S. V. Isakov, E. Jeffrey, Z. Jiang, D. Kafri, K. Kechedzhi, J. Kelly, P. V. Klimov, S. Knysh, A. Korotkov, F. Kostritsa, D. Landhuis, M. Lindmark, E. Lucero, D. Lyakh, S. Mandrà, J. R. McClean, M. McEwen, A. Megrant, X. Mi, K. Michielsen, M. Mohseni, J. Mutus, O. Naaman, M. Neeley, C. Neill, M. Y. Niu, E. Ostby, A. Petukhov, J. C. Platt, C. Quintana, E. G. Rieffel, P. Roushan, N. C. Rubin, D. Sank, K. J. Satzinger, V. Smelyanskiy, K. J. Sung, M. D. Trevithick, A. Vainsencher, B. Villalonga, T. White, Z. J. Yao, P. Yeh, A. Zalcman, H. Neven, J. M. Martinis, Quantum supremacy using a programmable superconducting processor. *Nature* **574**, 505–510 (2019).

2. Y. Wu, W.-S. Bao, S. Cao, F. Chen, M.-C. Chen, X. Chen, T.-H. Chung, H. Deng, Y. Du, D. Fan, M. Gong, C. Guo, C. Guo, S. Guo, L. Han, L. Hong, H.-L. Huang, Y.-H. Huo, L. Li, N. Li, S. Li, Y. Li, F. Liang, C. Lin, J. Lin, H. Qian, D. Qiao, H. Rong, H. Su, L. Sun, L. Wang, S. Wang, D. Wu, Y. Xu, K. Yan, W. Yang, Y. Yang, Y. Ye, J. Yin, C. Ying, J. Yu, C. Zha, C. Zhang, H. Zhang, K. Zhang, Y. Zhang, H. Zhao, Y. Zhao, L. Zhou, Q. Zhu, C.-Y. Lu, C.-Z. Peng, X. Zhu, J.-W. Pan, Strong quantum computational advantage using a superconducting quantum processor. *Phys. Rev. Lett.* **127**, 180501 (2021).

3. Q. Zhu, S. Cao, F. Chen, M.-C. Chen, X. Chen, T.-H. Chung, H. Deng, Y. Du, D. Fan, M. Gong, C. Guo, C. Guo, S. Guo, L. Han, L. Hong, H.-L. Huang, Y.-H. Huo, L. Li, N. Li, S. Li, Y. Li, F. Liang, C. Lin, J. Lin, H. Qian, D. Qiao, H. Rong, H. Su, L. Sun, L. Wang, S. Wang, D. Wu, Y. Wu, Y. Xu, K. Yan, W. Yang, Y. Yang, Y. Ye, J. Yin, C. Ying, J. Yu, C. Zha, C. Zhang, H. Zhang, K. Zhang, Y. Zhang, H. Zhao, Y. Zhao, L. Zhou, C.-Y. Lu, C.-Z. Peng, X. Zhu, J.-W. Pan, Quantum computational advantage via 60-qubit 24-cycle random circuit sampling. *Sci. Bull.* **67**, 240–245 (2022).

4. H.-S. Zhong, H. Wang, Y.-H. Deng, M.-C. Chen, L.-C. Peng, Y.-H. Luo, J. Qin, D. Wu, X. Ding, Y. Hu, P. Hu, X.-Y. Yang, W.-J. Zhang, H. Li, Y. Li, X. Jiang, L. Gan, G. Yang, L. You,

Z. Wang, L. Li, N.-L. Liu, C.-Y. Lu, J.-W. Pan, Quantum computational advantage using photons. *Science* **370**, 1460–1463 (2020).

5. H.-S. Zhong, Y.-H. Deng, J. Qin, H. Wang, M.-C. Chen, L.-C. Peng, Y.-H. Luo, D. Wu, S.-Q. Gong, H. Su, Y. Hu, P. Hu, X.-Y. Yang, W.-J. Zhang, H. Li, Y. Li, X. Jiang, L. Gan, G. Yang, L. You, Z. Wang, L. Li, N.-L. Liu, J. J. Renema, C.-Y. Lu, J.-W. Pan, Phase-programmable gaussian boson sampling using stimulated squeezed light. *Phys. Rev. Lett.* **127**, 180502 (2021).

6. J. Preskill, Quantum computing in the NISQ era and beyond. *Quantum* **2**, 79 (2018).

7. K. Bharti, A. Cervera-Lierta, T. H. Kyaw, T. Haug, S. Alperin-Lea, A. Anand, M. Degroote, H. Heimonen, J. S. Kottmann, T. Menke, W.-K. Mok, S. Sim, L.-C. Kwek, A. Aspuru-Guzik, Noisy intermediate-scale quantum algorithms. *Rev. Mod. Phys.* **94**, 015004 (2022).

8. S. Chen, J. Cotler, H.-Y. Huang, J. Li, The complexity of NISQ. *Nat. Commun.* **14**, 6001 (2023).

9. P. W. Shor, “Algorithms for quantum computation: Discrete logarithms and factoring” in *Proceedings 35th Annual Symposium on Foundations of Computer Science* (IEEE, 1994), pp. 124–134.

10. L. K. Grover, “A fast quantum mechanical algorithm for database search” in *Proceedings of the Twenty-Eighth Annual ACM Symposium on Theory of Computing* (ACM, 1996), pp. 212–219.

11. H. Buhrman, R. De Wolf, Complexity measures and decision tree complexity: A survey. *Theor. Comput. Sci.* **288**, 21–43 (2002).

12. A. C.-C. Yao, “Quantum circuit complexity” in *Proceedings of 1993 IEEE 34th Annual Foundations of Computer Science* (IEEE, 1993), pp. 352–361.

13. G. Brassard, Quantum communication complexity. *Found. Phys.* **33**, 1593–1616 (2003).

14. X. Gao, Z.-Y. Zhang, L.-M. Duan, A quantum machine learning algorithm based on generative models. *Sci. Adv.* **4**, eaat9004 (2018).

15. H.-Y. Huang, M. Broughton, J. Cotler, S. Chen, J. Li, M. Mohseni, H. Neven, R. Babbush, R. Kueng, J. Preskill, J. R. McClean, Quantum advantage in learning from experiments. *Science* **376**, 1182–1186 (2022).

16. S. Aaronson, L. Chen, "Complexity-theoretic foundations of quantum supremacy experiments" in *Proceedings of the 32nd Computational Complexity Conference* (ACM, 2017), pp. 1–67.

17. S. Aaronson, S. Gunn, On the classical hardness of spoofing linear cross-entropy benchmarking. *Theory Comput.* **16**, 1–8 (2020).

18. S. Boixo, S. V. Isakov, V. N. Smelyanskiy, R. Babbush, N. Ding, Z. Jiang, M. J. Bremner, J. M. Martinis, H. Neven, Characterizing quantum supremacy in near-term devices. *Nat. Phys.* **14**, 595–600 (2018).

19. A. M. Dalzell, N. Hunter-Jones, F. G. Brandao, Random quantum circuits anticoncentrate in log depth. *PRX Quantum* **3**, 010333 (2022).

20. A. Deshpande, P. Niroula, O. Shtanko, A. V. Gorshkov, B. Fefferman, M. J. Gullans, Tight bounds on the convergence of noisy random circuits to the uniform distribution. *PRX Quantum* **3**, 040329 (2022).

21. D. Aharonov, X. Gao, Z. Landau, Y. Liu, U. Vazirani, "A polynomial-time classical algorithm for noisy random circuit sampling" in *Proceedings of the 55th Annual ACM Symposium on Theory of Computing* (ACM, 2023), pp. 945–957.

22. Z. Cheng, M. Ippoliti, Efficient sampling of noisy shallow circuits via monitored unraveling. *PRX Quantum* **4**, 040326 (2023).

23. D. Hangleiter, J. Eisert, Computational advantage of quantum random sampling. *Rev. Mod. Phys.* **95**, 035001 (2023).

24. J. C. Napp, R. L. La Placa, A. M. Dalzell, F. G. Brandao, A. W. Harrow, Efficient classical simulation of random shallow 2D quantum circuits. *Phys. Rev. X* **12**, 021021 (2022).

25. S. D. Mishra, M. Fras-Pérez, R. Trivedi, Classically computing performance bounds on depolarized quantum circuits. *PRX Quantum* **5**, 020317 (2024).

26. A. M. Dalzell, N. Hunter-Jones, F. G. Brandão, Random quantum circuits transform local noise into global white noise. *Commun. Math. Phys.* **405**, 78 (2024).

27. X. Gao, M. Kalinowski, C.-N. Chou, M. D. Lukin, B. Barak, S. Choi, Limitations of linear cross-entropy as a measure for quantum advantage. *PRX Quantum* **5**, 010334 (2024).

28. Y. Zhou, E. M. Stoudenmire, X. Waintal, What limits the simulation of quantum computers? *Phys. Rev. X* **10**, 041038 (2020).

29. F. Pan, K. Chen, P. Zhang, Solving the sampling problem of the sycamore quantum circuits. *Phys. Rev. Lett.* **129**, 090502 (2022).

30. M.-H. Yung, X. Gao, Can chaotic quantum circuits maintain quantum supremacy under noise? arXiv:1706.08913 (2017).

31. S. Aaronson, A. Arkhipov, The computational complexity of linear optics. *Theory Comput.* **9**, 143–252 (2013).

32. D. Stilck França, R. Garcia-Patron, Limitations of optimization algorithms on noisy quantum devices. *Nat. Phys.* **17**, 1221–1227 (2021).

33. G. González-Garcia, R. Trivedi, J. I. Cirac, Error propagation in nisq devices for solving classical optimization problems. *PRX Quantum* **3**, 040326 (2022).

34. G. De Palma, M. Marvian, C. Rouzé, D. S. França, Limitations of variational quantum algorithms: A quantum optimal transport approach. *PRX Quantum* **4**, 010309 (2023).

35. X. Gao, L. Duan, Efficient classical simulation of noisy quantum computation. arXiv:1810.03176 (2018).

36. K. Noh, L. Jiang, B. Fefferman, Efficient classical simulation of noisy random quantum circuits in one dimension. *Quantum* **4**, 318 (2020).

37. M. Ben-Or, D. Gottesman, A. Hassidim, Quantum refrigerator. arXiv:1301.1995 (2013).

38. B. Fefferman, S. Ghosh, M. Gullans, K. Kuroiwa, K. Sharma, Effect of nonunital noise on random-circuit sampling. *PRX Quantum* **5**, 030317 (2024).

39. A. A. Mele, A. Angrisani, S. Ghosh, S. Khatri, J. Eisert, D. S. França, Y. Quek, Noise-induced shallow circuits and absence of barren plateaus. arXiv:2403.13927 (2024).

40. S. Wang, E. Fontana, M. Cerezo, K. Sharma, A. Sone, L. Cincio, P. J. Coles, Noise-induced barren plateaus in variational quantum algorithms. *Nat. Commun.* **12**, 6961 (2021).

41. Y. Shao, F. Wei, S. Cheng, Z. Liu, Simulating noisy variational quantum algorithms: A polynomial approach. *Phys. Rev. Lett.* **133**, 120603 (2024).

42. A. Morvan, B. Villalonga, X. Mi, S. Mandrà, A. Bengtsson, P. V. Klimov, Z. Chen, S. Hong, C. Erickson, I. K. Drozdov, J. Chau, G. Laun, R. Movassagh, A. Asfaw, L. T. A. N. Brandão, R. Peralta, D. Abanin, R. Acharya, R. Allen, T. I. Andersen, K. Anderson, M. Ansmann, F. Arute, K. Arya, J. Atalaya, J. C. Bardin, A. Bilmes, G. Bortoli, A. Bourassa, J. Bovaird, L. Brill, M. Broughton, B. B. Buckley, D. A. Buell, T. Burger, B. Burkett, N. Bushnell, J. Campero, H. S. Chang, B. Chiaro, D. Chik, C. Chou, J. Cogan, R. Collins, P. Conner, W. Courtney, A. L. Crook, B. Curtin, D. M. Debroy, A. D. T. Barba, S. Demura, A. Di Paolo, A. Dunsworth, L. Faoro, E. Farhi, R. Fatemi, V. S. Ferreira, L. F. Burgos, E. Forati, A. G. Fowler, B. Foxen, G. Garcia, E. Genois, W. Giang, C. Gidney, D. Gilboa, M. Giustina, R. Gosula, A. G. Dau, J. A. Gross, S. Habegger, M. C. Hamilton, M. Hansen, M. P. Harrigan, S. D. Harrington, P. Heu, M. R. Hoffmann, T. Huang, A. Huff, W. J. Huggins, L. B. Ioffe, S. V. Isakov, J. Iveland, E. Jeffrey, Z. Jiang, C. Jones, P. Juhas, D. Kafri, T. Khattar, M. Khezri, M. Kieferová, S. Kim, A. Kitaev, A. R. Klots, A. N. Korotkov, F. Kostritsa, J. M. Kreikebaum, D. Landhuis, P. Laptev, K.-M. Lau, L. Laws, J. Lee, K. W. Lee, Y. D. Lensky, B. J. Lester, A. T. Lill, W. Liu, W. P. Livingston, A. Locharla, F. D. Malone, O. Martin, S. Martin, J. R. McClean, M. McEwen, K. C. Miao, A. Mieszala, S. Montazeri, W. Mruczkiewicz, O. Naaman, M. Neeley, C. Neill, A. Nersisyan, M. Newman, J. H. Ng, A. Nguyen, M. Nguyen, M. Y. Niu, T. E. O'Brien, S. Omonije, A. Opremcak, A. Petukhov, R. Potter, L. P. Pryadko, C. Quintana, D. M. Rhodes, E. Rosenberg, C. Rocque, P. Roushan, N. C. Rubin, N. Saei, D. Sank, K. Sankaragomathi, K. J. Satzinger, H. F. Schurkus, C. Schuster, M. J. Shearn, A.

Shorter, N. Shutty, V. Shvarts, V. Sivak, J. Skruzny, W. C. Smith, R. D. Somma, G. Sterling, D. Strain, M. Szalay, D. Thor, A. Torres, G. Vidal, C. V. Heidweiller, T. White, B. W. K. Woo, C. Xing, Z. J. Yao, P. Yeh, J. Yoo, G. Young, A. Zalcman, Y. Zhang, N. Zhu, N. Zobrist, E. G. Rieffel, R. Biswas, R. Babbush, D. Bacon, J. Hilton, E. Lucero, H. Neven, A. Megrant, J. Kelly, I. Aleiner, V. Smelyanskiy, K. Kechedzhi, Y. Chen, S. Boixo, Phase transition in random circuit sampling. arXiv:2304.11119 (2023).

43. B. Ware, A. Deshpande, D. Hangleiter, P. Niroula, B. Fefferman, A. V. Gorshkov, M. J. Gullans, A sharp phase transition in linear cross-entropy benchmarking. arXiv:2305.04954 (2023).

44. S. Zhang, "Quantum strategic game theory" in *Proceedings of the 3rd Innovations in Theoretical Computer Science Conference* (ACM, 2012), pp. 39–59.

45. R. Jain, Y. Shi, Z. Wei, S. Zhang, Efficient protocols for generating bipartite classical distributions and quantum states. *IEEE Trans. Inf. Theory* **59**, 5171–5178 (2013).

46. S. Fiorini, S. Massar, S. Pokutta, H. R. Tiwary, R. de Wolf, "Linear vs. semidefinite extended formulations: Exponential separation and strong lower bounds" in *Proceedings of the Forty-Fourth Annual ACM Symposium on Theory of Computing* (ACM, 2012), pp. 95–106.

47. H. Fawzi, J. Gouveia, P. A. Parrilo, R. Z. Robinson, R. R. Thomas, Positive semidefinite rank. *Math. Program.* **153**, 133–177 (2015).

48. S. A. Vavasis, On the complexity of nonnegative matrix factorization. *SIAM J. Optim.* **20**, 1364–1377 (2010).

49. Y. Shitov, The complexity of positive semidefinite matrix factorization. *SIAM J. Optim.* **27**, 1898–1909 (2017).

50. J. Sikora, A. Varvitsiotis, Z. Wei, Minimum dimension of a Hilbert space needed to generate a quantum correlation. *Phys. Rev. Lett.* **117**, 060401 (2016).

51. Y. Shitov, Euclidean distance matrices and separations in communication complexity theory. *Discrete Comput. Geom.* **61**, 653–660 (2019).

52. N. Gillis, F. Glineur, On the geometric interpretation of the nonnegative rank. *Linear Algebra Its Appl.* **437**, 2685–2712 (2012).

53. L. Lin, Z. Chen, X. Lin, Z. Wei, All pure bipartite entangled states can be semi-self-tested with only one measurement setting on each party. arXiv:2306.07755 (2023).

54. Z. Chen, L. Lin, X. Lin, Z. Wei, P. Yao, The generations of classical correlations via quantum schemes. *IEEE Trans. Inf. Theory* **70**, 4160–4169 (2024).
